# The Genetics and Epigenetics of Ventricular Arrhythmias in Patients Without Structural Heart Disease

**DOI:** 10.3389/fcvm.2022.891399

**Published:** 2022-06-15

**Authors:** Mengru Wang, Xin Tu

**Affiliations:** Key Laboratory of Molecular Biophysics of the Ministry of Education, Center for Human Genome Research, College of Life Science and Technology, Huazhong University of Science and Technology, Wuhan, China

**Keywords:** gene, pathogenesis, non-structural ventricular arrhythmias with genome, ventricular arrhythmias, non-structural heart disease

## Abstract

Ventricular arrhythmia without structural heart disease is an arrhythmic disorder that occurs in structurally normal heart and no transient or reversible arrhythmia factors, such as electrolyte disorders and myocardial ischemia. Ventricular arrhythmias without structural heart disease can be induced by multiple factors, including genetics and environment, which involve different genetic and epigenetic regulation. Familial genetic analysis reveals that cardiac ion-channel disorder and dysfunctional calcium handling are two major causes of this type of heart disease. Genome-wide association studies have identified some genetic susceptibility loci associated with ventricular tachycardia and ventricular fibrillation, yet relatively few loci associated with no structural heart disease. The effects of epigenetics on the ventricular arrhythmias susceptibility genes, involving non-coding RNAs, DNA methylation and other regulatory mechanisms, are gradually being revealed. This article aims to review the knowledge of ventricular arrhythmia without structural heart disease in genetics, and summarizes the current state of epigenetic regulation.

## Introduction

The vast majority of ventricular arrhythmias occurs in structurally diseased hearts, however, a proportion of patients with ventricular tachycardia is free of cardiac structure alterations (1). Ventricular arrhythmias without structural heart disease mainly includes monomorphic ventricular tachycardia classified by location of origin, polymorphic ventricular tachycardia dominated by primary hereditary arrhythmia syndrome, and ventricular fibrillation, i.e., Brugada syndrome (BrS), congenital long QT syndrome (LQTS), short QT syndrome (SQTS), catecholaminergic polymorphic ventricular tachycardia (CPVT) (2, 3). The clinical presentations vary, including palpitations, vertigo, syncope, seizure-like activity and sudden cardiac death. There is no obvious cardiac structural change in patients without structural heart disease, but it may also be due to the lack of detection of pathological changes in the existing technology or focal lesions in the heart. Especially in BrS, structural abnormality has also become one of the pathogenesis, however, due to the restrictions on the acquisition of human samples, structural detection is mostly carried out in patients with severe symptoms. The prominent role of genetics in the pathogenesis of the disease remains of interest (4, 5). With the development of detection technology, we may gain a greater understanding of the mechanisms involved in this disease.

The heart has a set of well-established electrical conduction systems, and the tissues and cells coordinate with each other to make the heart contract and relax in an orderly way, and pump blood to all parts of the body. The action potential (AP) of human ventricular myocytes is composed of depolarization and repolarization, and is subdivided into five stages, 0 and 1, 2, 3, 4 (6). The flow balance of potassium, sodium, calcium and other ions is essential for the normal beating of the heart. Cardiomyocyte depolarization induced by electrotonic coupling between adjacent cardiomyocytes drives phase 0 initiation, the sodium channel is activated, the sodium ion enters the membrane rapidly, resulting in depolarization (phase 0) (7). Subsequently, Majority of sodium channels are rapidly inactivated and L-type calcium channels are activated. At the same time, the repolarization current generated by potassium channels and Na^+^/Ca^2+^ exchangers, namely the instantaneous outward potassium current (Ito) and outward I_NCX_ reduce the membrane potential (phase 1) (8). the sodium current of slowly inactivated sodium channels, mostly inactive calcium channels current (I_Ca, L_) and the sodium-potassium exchanger current operating in forward mode reach a balance with delayed rectifier potassium current (I_K_), forming a plateau phase on ECG (phase 2). While the action potential occurs, the influx of calcium ions triggers a further release of calcium ions from the sarcoplasmic reticulum, resulting in intracellular concentration transient elevation, causing muscle contraction, namely cardiac excitation-contraction (E-C) coupling (9). Subsequently, the calcium ion channel is gradually inactivated. The ion current at this time is mainly the outward current of I_K_ and I_K1_, and the more negative the intramembrane potential, the more rapid efflux of the potassium ion, which leads to the acceleration of the repolarization until the repolarization is completed (phase 3). The potential is stable at the resting potential level, and the ion pump pumps out the ions pumped into the cell during the action potential. The Na^+^/ K^+^ pump can pump the Na^+^ in the cell out of the cell and pump K^+^ into the cell at the same time. Intracellular Ca^2+^ is transported extracellular via the Na^+^/ Ca^2+^ exchanger and the Ca^2+^ pump (phase 4) (10). The abnormality caused by the mutation of ion channel protein coding gene will cause the disorder of electrical signal.

Triggered activity, abnormal automaticity, and re-entry are the three mechanisms of ventricular arrhythmias, in ventricular arrhythmias without structural heart disease, usually due to trigger activity (3). Triggered activity that occurs in phase 2 and early phase 3 is called early afterdepolarization (EAD), in late phase 3 and phase 4 are called delayed after-depolarization (DAD) (11), The trigger activity is generated by the membrane depolarisation induced by the I_NCX_ and/or Ica,l (12, 13). Automaticity refers to the spontaneous depolarization of phase 4 membrane potential of the cells with pacemaking function, and the action potential is generated after reaching the threshold potential. Abnormal automaticity is attributed to decreased I_K1_ and/or enhanced I_f_ (mainly slow inward sodium current, causing automatic membrane depolarization) (14). Once I_K1_ is inhibited, the membrane potential cannot reach the resting potential, which may lead to the generation of abnormal inward currents, such as I_f_, causing abnormal automaticity. Re-entry refers to a cardiac impulse that repeatedly runs and activates the cardiac muscle surrounding around the center of anatomical or functional disorders, usually the pathogenesis of structural ventricular arrhythmias, but specific arrhythmias induced by EAD (Torsades de Pointes in LQTS) or DAD (bidirectional ventricular tachycardia in CPVT) may be caused by re-entry involving fascicles of the Purkinje system (3).

In respect to etiology, ventricular arrhythmias in patients without structural heart disease are mostly due to ion channel disorders, including various inherited arrhythmia syndromes and ventricular arrhythmias caused by unknown causes. Besides genetic studies in classical, the effects of epigenetics on ventricular arrhythmias are also being explored, which makes this part that is not yet understood gradually revealed. We searched the PubMed database using the terms “idiopathic ventricular arrhythmias”, “genetics”, “epigenetics”, and “DNA methylation” up to 2021 for articles on ventricular arrhythmias and present the genetics and epigenetics of ventricular arrhythmias in non-structural heart disease in this review. Table 1 is a brief description.

**Table 1:** A brief description of the genetics and epigenetics of ventricular arrhythmias in non-structural heart disease.

	**Causative factor**	**Description**	**References**
Genetics	Monogenic factors	Among the monogenic causative factors, mutations in the sodium channel-encoding gene SCN5A, the potassium channel-encoding genes KCNQ1 and KCNH2, and the calcium channel-encoding gene RYR2 cause the majority of ventricular arrhythmias, while some cases are caused by rare variants in other ion channel and structural genes. In addition, the role of somatic mutations has been identified.	(15–18)
	Polygenic factors	The importance of polygenic factors for ventricular arrhythmias is highlighted by the heterogeneity of causative genes across patients with ventricular arrhythmias and the impact of the accumulation of mutations in multiple genes on the severity of the clinical phenotype. The concept of genetic modifier has been proposed and a recent GWAS analysis validated the link between cumulative mutational effects and the BRS clinical phenotype.	(19–22)
Epigenetics	Non-coding RNA	The research on ventricular arrhythmias without structural heart disease mainly focuses on the regulation of miRNAs on the transcription of genes encoding ion channels such as SCN5A and SCN1B. Circular RNA may serve as a marker for disease progression.	(23–25)
	DNA methylation	DNA methylation usually plays a repressive role in gene transcription. For example, SCN5A promoter hypermethylation levels enhance SCN5A expression in cardiac tissue. In addition, it plays an important regulatory role in gene imprinting.	(26, 27)
	Histone modifications	Histone modifications in the heart have mostly been studied for methylation and acetylation, which are linked to gene transcriptional activation or repression and may play a role in the formation of transmural electrophysiological gradients in the ventricle.	(28–30)
	Genomic imprinting	The methylation level of the long non-coding RNA KCNQ1OT1, which is related to a prolonged QTC interval, affects the expression of the imprinted gene KCNQ1 and may contribute to female predominance and transmission distortion in LQTS.	(31–33)
	Three-dimensional (3D) genome architecture	The ordered chromatin spatial structure allows interactions between functional elements within the topological domains to regulate gene transcription, such as the interaction of enhancers with promoters. The 3D genome architecture study offers a fresh look at the link between SNPs and ventricular arrhythmias discovered by GWAS.	(34–37)

## Genetic Factors of Ventricular Arrhythmias Without Structural Heart Disease

The advancement of genetic technology has brought a deeper understanding of the genetic factors of the disease. It is a common Mendelian genetic phenomenon that a rare variant of a single gene has a significant impact on protein function and causes disease. However, the incomplete penetrance in the family and the frequency of pathogenic mutations in the normal population have begun to make people think that disease is not only caused by the mutation of a certain gene locus, but that the cumulative pattern of multi-site interaction can also cause the occurrence of disease. Here, we discussed the single-gene and multi-gene factors that cause ventricular arrhythmias and summarized the single gene factors in Table 2.

**Table 2:** Genes associated with ventricular arrhythmias in non-structural heart disease.

**Gene**	**Protein (UniProtKB)**	**Aliases**	**Functional effect**	**Symptom**	**Frequency, %**	**References**
**Sodium channel-related genes**						
SCN5A	Sodium channel protein type 5 subunit alpha	Nav1.5/LQT3/VF1	I_Na_↑	LQTS	5–10%	(15)
			I_Na_↓	BrS	20–25%	(15)
SCN1B	Sodium channel subunit beta-1		I_Na_↓	BrS	Rare	(38)
SCN2B	Sodium channel subunit beta-2		I_Na_↓	BrS	Rare	(39, 40)
SCN3B	Sodium channel subunit beta-3		I_Na_↓	BrS	Rare	(41)
SCN4B	Sodium channel subunit beta-4	LQT10	I_Na_↑	LQTS	Rare	(42)
GPD1L	Glycerol-3-phosphate dehydrogenase 1-like protein	GPD1-L	I_Na_↓	BrS	Rare	(43)
RANGRF	Ran guanine nucleotide release factor	RANGNRF/MOG1	I_Na_↓	BrS	Rare	(44, 45)
SCN10A	Sodium channel protein type 10 subunit alpha	Nav1.8	I_Na_↓	BrS	~10%	(46)
**Potassium channel-related genes**						
*Hyperpolarization-activated cyclic nucleotide–gated (HCN) channels*						
HCN4	Potassium/sodium hyperpolarization-activated cyclic nucleotide-gated channel 4		I_f_↓	IVT	Rare	(47)
*transient outward potassium current channels*						
KCND3	Potassium voltage-gated channel subfamily D member 3	Kv4.3	I_to_↑	BrS	Rare	(48)
KCNE3	Potassium voltage-gated channel subfamily E member 3	MiRP2/HOKPP	I_to_↑	BrS	Rare	(49)
KCNE5	Potassium voltage-gated channel subfamily E regulatory beta subunit 5	MiRP4	I_to_↑	BrS	Rare	(50)
*Slowly activating delayed rectifier potassium current channels*						
KCNQ1	Potassium voltage-gated channel subfamily KQT member 1	KVLQT1/Kv7.1/LQT1	I_Kr_↓	LQTS	30–35%	(51)
			I_Kr_↑	SQTS	Unknown	(52)
KCNE1	Potassium voltage-gated channel subfamily E member 1	MinK 2/LQT5	I_Kr_↓	LQTS	Rare	(53)
*Rapidly activating delayed rectifier potassium current channels*						
KCNH2	Potassium voltage-gated channel subfamily H member 2	HERG/Kv11.1/ERG-1/LQT2	I_Kr_↓	LQTS	25–30%	(54)
			I_Kr_↑	SQTS	Unknown	(55)
KCNE2	Potassium voltage-gated channel subfamily E member 2	MiRP1/LQT6	I_Kr_↓	LQTS	Rare	(56)
*Inwardly rectifying potassium (Kir) channels*						
Inwardly rectifying potassium channels						
KCNJ2	Inward rectifier potassium channel 2	Kir2.1/LQT7	I_K1_↑	SQTS	Unknown	(57)
			I_K1_↓	LQTS	Rare	(58)
G protein-coupled, inwardly rectifying potassium channels						
KCNJ5	G protein-activated inward rectifier potassium channel 4	Kir3.4/GIRK4/LQT13	I_KACh_↓	LQTS	Rare	(59)
ATP-sensitive potassium channels						
KCNJ8	ATP-sensitive inward rectifier potassium channel 8	Kir6.1	I_K−ATP_↑	BrS	Rare	(60)
ABCC9	ATP-binding cassette sub-family C member 9	SUR2	I_K−ATP_↑	BrS	Rare	(61)
**Calcium channel-related genes**						
CACNA1C	Voltage-dependent L-type calcium channel subunit alpha-1C	Cav1.2/LQT8	I_Ca, L_↑	LQTS	1–2%	(62)
			I_Ca, L_↓	BrS	1–2%	(63)
CACNB2	Voltage-dependent L-type calcium channel subunit beta-2	CACNLB2	I_Ca, L_↓	BrS	1–2%	(63)
CACNA2D1	Voltage-dependent calcium channel subunit alpha-2/delta-1	CACNL2A	I_Ca, L_↓	BrS	Rare	(64)
RYR2	Ryanodine receptor 2	ARVC2/ARVD2	Aberrant calcium handling	CPVT	55–60%	(65)
			Aberrant calcium handling	IVF	Rare	(66)
CASQ2	Calsequestrin-2		Aberrant calcium handling	CPVT	<5%	(67)
TRDN	Triadin	TDN/TRISK/CPVT5	Aberrant calcium handling	CPVT	1–2%	(68)
			I_Ca, L_↑	LQTS	1–2%	(69)
CALM1~3	Calmodulin-1~3	CaMI/CaMII/CaMIII	I_Ca, L_↑	LQTS	Rare	(70)
			Aberrant calcium handling	CPVT	Rare	(70)
**Other related genes**						
SNTA1	Alpha-1-syntrophin	LQT12/SNT1	I_Na_↑	LQTS	Rare	(71)
SLMAP	Sarcolemmal membrane-associated protein	SLAP	I_Na_↓	BrS	Rare	(72)
PKP2	Plakophilin-2	ARVD9	I_Na_↓	BrS	Rare	(73)
ANK2	Ankyrin-2	LQT4	Abnormal coordination of multiple functionally related ion channels and transporters	LQTS	Rare	(74)
CAV3	Caveolin-3		I_Na_↑I_Ca, L_↑I_K_↓I_to_↓	LQTS	Rare	(75)
TECRL	Trans-2,3-enoyl-CoA reductase-like	TERL	Aberrant calcium handling	Mixed phenotype of CPVT and LQTS	Rare	(76)
SLC4A3	Anion exchange protein 3	AE3/SLC2C	Phi↑	SQTS	Unknown	(77)
TRPM4	Transient receptor potential cation channel subfamily M member 4		uncertain	BrS, LQTS	Rare	(78)
**Genes specific to IVF**						
DPP6	Dipeptidyl aminopeptidase-like protein 6	DPPX/DPP VI/VF2	I_to_↑	IVF	Unknown	(79)
IRX3	Iroquois-class homeodomain protein IRX-3	IRX-1/IRXB1	I_Na_↓	IVF	Unknown	(80)
**Somatic mutation genes**						
GNAI2	Guanine nucleotide-binding protein G(i) subunit alpha-2	GNAI2B/GIP	cAMP↑	RVOT-VT	Rare	(18)
ADORA1	Adenosine receptor A1	A1AR	unknown	RVOT-VT	Rare	(81)
GNAS	Guanine nucleotide-binding protein G(s) subunit alpha isoforms short	GNAS1/NESP	I_Ca, L_↑	RVOT-VT	Rare	(82)

*BrS, Brugada syndrome; LQTS, long QT syndrome; SQTS, short QT syndrome; CPVT, catecholaminergic polymorphic ventricular tachycardia; IVF, idiopathic ventricular fibrillation; IVT, idiopathic ventricular tachycardia; RVOT-VT, right ventricular outflow tract ventricular tachycardia; ↑, increased and/ or enhanced; ↓, decreased and/ or weakened*.

### Monogenic Factors

#### Sodium Ion Channels

Mutations in the gene encoding voltage-gated sodium channels can disrupt sodium channels, cause abnormal sodium current flow and trigger ventricular arrhythmias, due to the significant role of voltage-gated sodium channels in action potentials (15). Alterations in sodium current can cause several types of cardiac disease, including LQTS, BrS, isolated (progressive) conduction defect, atrial fibrillation, sick sinus syndrome, dilated cardiomyopathy and multifocal ectopic premature Purkinje-related complexes (15). In ventricular arrhythmias without structural heart disease, enhanced sodium currents are usually associated with LQTS, whereas diminished sodium currents are associated with BrS (15, 83). Several sodium channel-related pathogenic genes have been identified in ventricular arrhythmias without structural heart disease, including SCN5A, SCN1B, SCN2B, SCN3B, SCN4B, GPD1L, RANGRF, SCN10A, among which SCN5A is the most reported (Table 2). SCN5A encodes Na_v_1.5, which is the alpha subunit of voltage-gated sodium channels. As the main functional subunit of sodium ion channels, it provides activity for the channel. Na_v_1.5 is responsible for the influx of sodium ions and plays a major role during membrane depolarization, as well as the sodium current in the repolarization and refractory period. SCN5A Mutation can be divided into gain-of-function and loss-of-function mutation, leading to an increase or decrease in sodium ion influx and an acceleration or delay in channel inactivation, responsible for about 5–10% of LQTS patients and ~30% of patients with BrS, respectively (15, 16).

In addition to Nav1.5 that directly affects sodium currents, some proteins can achieve indirect regulation of sodium currents by modulating Nv1.5, have been associated with BrS and LQTS. although their clinical relevance may be limited. SCN1B, SCN2B, and SCN3B encode voltage-gated sodium channel β subunits. Mutations can decrease Na_v_1.5 cell surface expression and reduce sodium current, leading to BrS (38, 39, 41). SCN4B also encodes sodium channel β subunit, can cause LQTS in a minority of cases. Consistent with the molecular/electrophysiological phenotype previously displayed by LQTS, compared to wild type, the mutation L179F of SCN4B leads to an increase of late sodium current (42). GPD1L is SCN5A regulatory proteins, links redox state to cardiac excitability by PKC-dependent phosphorylation of the sodium channel (84). The GPD1L A280V mutation reduces SCN5A membrane expression, decreases inward Na^+^ current, leading to BrS (43). RANGRF (MOG1) is a co-factor of Na_v_1.5, which plays a potential role in the regulation of Na_v_1.5 expressions and trafficking. The dominant-negative mutations in RANGRF can disrupt the transport of Na_v_1.5 to the membrane, resulting in a reduction of I_Na_ and causing clinical manifestations of BrS. Silencing RANGRF can reduce I_Na_ density, in addition, replacing E83D, D148Q, R150Q, and S151Q could disrupt the interaction of RANGRF with Na_v_1.5 and significantly impair the trafficking of Na_v_1.5 to the cell surface, indicating that RANGRF may play an important role in the expression of Na_v_1.5 channel on the cell surface (44, 45). SCN10A encodes Na_v_1.8, a voltage-gated sodium channel like Na_v_1.5. Both SCN10A and SCN5A are located on chromosome 3p22.2. Co-expression of SCN10A-WT and SCN5A can increase sodium channel current, while SCN10A mutants (R14L and R1268Q) can cause loss of Na_v_1.5 current function, which may be the genetic basis of BrS caused by SCN10A. However, most of the mutations associated with SCN10A are not rare variants, are relatively frequent in the population (46).

#### Potassium Ion Channels

The potassium ion currents associated with ventricular arrhythmias include transient outward potassium current (I_to_), delayed rectifier potassium current (I_K_), inward rectifier K^+^ current (I_K1_), and hyperpolarization-activated currents (I_h_). Dysfunction of potassium ion channels can also cause changes in ion balance, causing arrhythmia (Table 2).

Hyperpolarization-activated cyclic nucleotide–gated (HCN) channels are responsible for pacing current in neurons and cardiomyocytes, in which HCN4 encode a member of the hyperpolarization-activated cyclic nucleotide–gated (HCN) channels showing slow activation and inactivation kinetics and is the highest expressed isotype in sinoatrial node (SAN) muscle cells, necessary for cardiac pacing (85). HCN4 gene mutation was detected in patients with idiopathic ventricular arrhythmias, which resulted in the decrease of pacemaker (I_f_) current (85, 86).

The repolarization of the ventricles is spatially heterogeneous, largely dependent on the gradient of potassium current, especially I_to_ (87). Under normal circumstances, the density of I_to_ in the epicardium is higher than the endocardium and the initial repolarization of the epicardium is earlier than the endocardium, generating a transmural voltage gradient that plays an important role in synchronizing repolarization (87). Gain of function of I_to_ is related to BrS. I_to_ channel is composed of α subunit and β subunit, with the α subunit K_v_4.3 encoded by KCND3, and the negatively regulated β-subunit MiRP2 encoded by KCNE3. It is a voltage-gated, calcium-independent potassium (K_v_) current that can be quickly activated and inactivated, mainly responsible for the initial repolarization phase of the cardiac action potential (88), KCND3 mutations are known to cause BrS. The mutation KCND3 Arg431His (c.1292G>A) detected in BrS patients does not affect the mRNA and total protein expression level of K_v_4.3, but increase the membrane protein expression of K_v_4.3 and up-regulate the transient outward potassium current (48). Besides, at the same time, KCNE3 mutation may be the basis of the development of BrS. The co-transfection of the KCNE3 gain-of-function mutation R99H with KCND3 causes a significantly increased current intensity compared to WT KCNE3+KCND3 (49). Actually, the KCNE family encodes five isotypes in the human genome. In addition to KCNE3, KCND3 can be assembled with multiple KCNE subunits, of which KCNE5 can regulate I_to_, showing a correlation with BrS and idiopathic ventricular fibrillation (50). KCNE5 is located on the X chromosome and when coexpressed with KCND3, the brugada-associated KCNE5 mutations upregulate I_to_ compared to the wild type (50).

There are two types of delayed rectifier K^+^ current, slowly activating delayed rectifier K^+^ current (I_Ks_) and rapidly activating delayed rectifier K^+^ current (I_Kr_). Its enhancement is related to SQTS, whereas weakening is related to LQTS (89). The outward K^+^ current of I_Ks_ channel is one of the main repolarizing potassium currents in the human heart, which helps to terminate cardiac action potential. The channel is composed of the pore-forming α subunit encoded by KCNQ1 (also known as K_v_7.1 or K_v_LQT1) and the β subunit encoded by KCNE1 (90, 91). Cardiac KCNQ1/KCNE1 channel mutations are the most common cause of inherited long-QT syndromes (92). Loss-of-function mutations of KCNQ1 decrease I_Ks_ current density, leading to LQTS, and there are also mutations that are gain-of-function mutations increase I_Ks_, leading to SQTS (93, 94). Mutations in KCNE1 gene cause reduced I_Ks_ current density, manifested as loss of function, which can lead to LQTS (53). However, a recent study has indicated that QTc Interval Prolongation (>460 ms) was not observed in most people carrying KCNE1 loss-of-function mutations (95). The rapidly activating delayed rectifier potassium channel is encoded by KCNH2 and KCNE2, which produces only a small outward current during membrane depolarization, however, after the membrane is repolarized, the channel quickly recovers from the inactivation state to the open state, producing a recovery current before the slow channel deactivation. KCNH2 gene encodes the pore-forming α subunit of voltage-gated K^+^ channel K_v_11.1, commonly referred to as Herg. Approximately 90% of LQT- associated KCNH2 mutations reduced I_Kr_ by reducing K_v_11.1, responsible for 25–30% of LQTS cases channel synthesis or trafficking (54). Gain-of-function mutations are the genetic basis of SQTS, which increase the repolarization current activated in the early stages of AP, resulting in a shortening of the action potential and the QT interval (55). KCNE2 encodes MiRP1, an auxiliary beta-subunit. Compared with the wild-type channel, the mutant HERG/MiRP1 (V65M) channel detected in LQTS patients has a shorter current inactivation time, which may reduce the I_Kr_ current density of cardiomyocytes, weakening the cardiomyocytes' ability to repolarize sudden membrane depolarization (56). It has also been reported that overexpression of wild-type KCNE2 can rescue the phenotype caused by KCNH2 mutation, facilitating the transport of K_v_11.1 channel protein and cell surface expression, significantly increasing the mutation current (96).

Inwardly rectifying potassium (Kir) channels allow potassium ions to move more easily into rather than out of the cell. The inwardly rectifying potassium channels gene associated with ventricular arrhythmia has KCNJ2, KCNJ5, KCNJ8, and ABCC9 (Table 2). I_K1_ plays an important role in stabilizing the resting membrane potential, regulating excitability and causing the final repolarization of atrial and ventricular action potential. Like delayed rectifier potassium channel, its enhancement is related to SQTS and reduction is related to LQTS, while ATP-sensitive potassium channel current (I_K−ATP_) enhancement will lead to BrS. One of the molecular basis is Kir2.1, which is coded by KCNJ2. Gain-of-function KCNJ2 mutations have been found in SQTS patients, resulting in increased I_K1_, accelerated ventricular repolarization and shortened QT interval (57, 97). There are also reports in the literature that loss-of-function mutations can cause LQTS (58). Mutations in the Inwardly rectifying potassium channels Kir2.1 encoded by KCNJ2 causing loss of function are associated with a rare clinical phenotype called Andersen-Tawil syndrome (ATS), which contains pleomorphic ventricular tachycardia (98). KCNJ5 encodes Kir3.4, a subunit of the voltage-gated potassium channel, which is controlled by G proteins and combination with Kir3.1, responsible for acetylcholine-activated potassium channel current (I_KACh_) (59). The KCNJ5 mutation results in defects in channel trafficking and a reduction in the I_KACh_, which is considered to be the pathogenic gene of LQTS (59). ATP-sensitive potassium channel (K_ATP_) is one kind of inwardly rectifying channel composed of the pore forming subunits and the regulatory subunits. Kir6.1 encoded by KCNJ8 and SUR2 encoded by ABCC9 are both subunits of ATP-sensitive potassium channels (99). KCNJ8 mutation can reduce the sensitivity of K_ATP_ channels to ATP, resulting in enhanced I_K−ATP_ function, which may lead to BrS (60, 100). Gain-of-function mutation of ABCC9 has also been reported as a possible pathogenic site for BrS, however, the relevance of pathogenic requires further confirmation (61).

#### Calcium Ion Channels

Voltage-gated L-type calcium channel (LTCC) is the main channel mediating influx of calcium ions into cells in repolarization stage, and in addition, NCX, an ion transport protein, can mediate the exchange cycle of sodium ions and calcium ions with a coupling ratio of 3:1 (101). Calcium entering the cell through these two distinct mechanisms triggers the release of calcium from the sarcoplasmic reticulum. Voltage-gated L-type calcium channel consists of four subunits α1 subunit, auxiliary β subunit, α2δ subunit, and γ subunit. The four subunits of voltage-gated L-type calcium channels which are, respectively, coded by CACNA1C or CACNA1D, CACNB2, CACNA2D, and CACNG (102, 103). Among them, the mutant genes that have been reported to cause ventricular arrhythmia are CACNA1C, CACNB2, CACNA2D (Table 2). CACNA1C encodes Ca_v_1.2 pore-forming α1 subunit. In general, the enhancement of I_Ca, L_ is related to LQTS, on the contrary causes BrS. Timothy syndrome (TS), which manifests as severe LQTS and multiorgan dysfunction, is mainly caused by gain-of-function mutations located in CACNA1C alternative splicing exon 8 which leads to almost complete loss of voltage-related channel inactivation, resulting in maintained inward Ca^2+^ current, and prolonged Ca^2+^ current that delay cardiomyocyte repolarization (104–106). A few cases are caused by mutations within exon 9 and exon 38 of CACNA1C (107, 108). Gain-of-function mutations in CACNA1C at positions other than exon 8 can cause LQTS with non-Timothy syndrome symptoms (62, 109). The phenotype caused by the loss-of-function mutations of the cardiac L-type calcium channel α1 and β2b subunits is similar to calcium channel blockers, leading to reduced I_Ca, L_, resulting in BrS (63, 110, 111). Subsequent studies identified CACNA2D1 as a novel BrS/SQTS susceptibility gene (64, 112). In addition, it is also reported that KCNE2 can affect I_Ca, L_ by regulating Ca_v_1.2 (113).

The main function of the sarcoplasmic reticulum (SR) is to store calcium in striated muscle. The genes involved in the release of calcium from the sarcoplasmic reticulum are RYR2, CASQ2, TRDN, CALM1, CALM2, and CALM3, which are pathogenic genes of CPVT (Table 2) (65, 114). Among them, TRDN, CALM1, CALM2, CALM3 mutations can also cause LQTS. RYR2 encodes ryanodine receptors (RyR2), responsible for the rapid release of calcium ions from the sarcoplasmic/endoplasmic reticulum (SR/ER) into the cytoplasm. RYR2 mutations may be either loss or gain of function and this may be related with different clinical phenotypes. Approximately 60% of patients with CPVT carry RYR2 mutations, and the main pathogenic mechanism of gain-of-function mutations is increased spontaneous RyR2 opening and pathological calcium release during diastole (17, 65, 115). The RyR2 mutation associated with idiopathic ventricular fibrillation confirmed as a loss-of-function mutation exhibiting Ca^2+^ release deficiency (66). However, a recent study identified a loss-of-function mutation D3291V, which markedly reduced luminal Ca^2+^ sensitivity, and blunted response to adrenergic stimulation, also exhibiting a CPVT phenotype (116). The relationship between gain-of-function or loss-of-function and the clinical phenotype still needs to be elucidated (117). The calsequestrin encoded by CASQ2 is a calcium-binding protein that regulates the amount of calcium released from the SR during excitation-contraction coupling by buffering the calcium in the SR (118, 119). Triadin (TRD) encoded by TRDN is located on the sarcoplasmic reticulum, forms a complex with ryanodine receptors, calsequestrin and junctin, regulating the storage and release of calcium ions (120). Triadn anchors calsequestrin to junctional SR membrane and stabilizes the structure of Ca 2+ release units (121). TRDN deficiency leads to significantly reduced protein levels of RyR2, calsequestrin, and junctin, impaired coupling efficiency between LTCC and RyR2, reduced SR calcium release and calcium-dependent inactivation of LTCC, resulting in defective cardiac excitation-contraction coupling (121, 122). Similarly, Triadin mutations are thought to result in reduced protein levels, which in turnincreased calcium currents and prolonged cardiac action potentials, and increased spontaneous calcium release events caused by cellular and SR calcium overload, which is the basis of TRDN mutation leading to CPVT and LQTS (69, 123). CALM1~3 encodes Calmodulin, which is Ca^2+^ sensor, signal-transducing protein. Calmodulin binds to RyR2 and LTCC, plays a key role in the calcium-dependent inactivation of the L-type calcium channel Ca_v_1.2 and the timely closure of the myocardial sarcoplasmic reticulum calcium release channel RyR2. Calcium binding to calmodulin inactivates the LTCC channel, namely calcium-dependent inactivation (124). The mutations associated with LQTS are mainly due to weakening of the combination of calmodulin and calcium, completely eliminating the calcium-dependent inactivation. On the other hand, the binding of calmodulin to RyR2 can inhibit the release of calcium from SR during diastole. Mutations associated with CPVT mainly lead to dysregulation of RyR2 calcium release (70, 125).

#### Others

The protein encoded by SNTA1 is a component of the Na_v_1.5 channel macromolecular complex, interacting with the pore-forming α subunit (126). Gain-of-function SNTA1 mutations can affect Na_v_1.5 gating kinetics, leading to the LQTS phenotype (71). SLMAP encodes the sarcolemmal associated protein located in T-tubules and sarcoplasmic reticulum. SLMAP mutation may cause BrS by regulating the intracellular transport of Na_v_1.5 channel (72). PKP2 encodes Plakophilin-2, a desmosomal protein. Loss and/or changes in the Plakophlin-2 structure in the heart desmosomes can impair the interaction between myocardial cells and cause myocardial rupture, especially in response to mechanical stress (127). Mutations in PKP2 have been associated with BrS, and the deletion of PKP2 can lead to a decrease in sodium current and Na_v_1.5 at the site of cell contact (73). Ankyrins bind to spectrins, connects the plasma membrane with the actin cytoskeleton, maintains mechanical strength and regulates the excitability of various cells. The ankyrin family contains three members: ankyrin-R, ankyrin-B and ankyrin-G, are encoded by ANK1, ANK2 and ANK3, respectively (128). Loss-of-function mutation (E1425G) in ankyrin-B (also known as ankyrin 2) can lead to dominant long QT arrhythmia (74, 129). CAV3 encodes a scaffold protein for the cavern in cardiomyocytes and participates in the separation of ion channels. CAV3 mutations are related to LQTS, which can prolong repolarization times by enhancing the late sodium current, enhancing the peak value of L-type calcium current, slowing down deactivation, reducing delayed rectifier potassium current and transient outward potassium current (75). TECRL (TERL) is an oxidoreductase enzyme localized to the endoplasmic reticulum. Patients with TECRL pathogenic variants manifest a specialized mixed phenotype of CPVT and LQTS, which is an autosomal recessive disease. Functional experiments have shown that homozygous pathogenic variants can lead to reduced levels of RYR2 and CASQ2 proteins, and reduced calcium storage of sarcoplasmic reticulum and aberrant calcium handling (76).

SLC4A3 (AE3) is an electroneutral Cl^−^/${\text{HCO}}_{3}^{-}$ exchanger (130). Studies have found that reducing intracellular pH (PHi) could prolong QT interval; the regulation of chloride ion conductance in cardiomyocytes can also change action potential duration. In zebrafish, SLC4A3 mutations can lead to transport defects, reduce Cl,HCO3-exchange over the cell membrane, increase pHi and reduce the QT duration, which may be another development mechanism of SQTS (77). The protein encoded by TRPM4 is a transmembrane N-glycosylated ion channel, a non-selective channel activated by intracellular calcium, permeable to monovalent cations. At present, Trpm4 mutations have been detected in both BrS and LQTS cohort. Miraculously, gain-and loss-of-function variants of TRPM4 channels can cause similar phenotypes (78).

Idiopathic ventricular fibrillation is an exclusive disease that requires excluding the presence of the ventricular fibrillation substrate and specific diseases, including structural heart disease and primary arrhythmia syndrome (131). At present, most genes related to idiopathic ventricular fibrillation overlap with the pathogenic genes of hereditary ventricular arrhythmias, such as CALM1~3, RYR2, TRDN, CACNA1C, SCN5A, KCNE5 (50, 125, 132–137). However, there are also specific causative genes in the idiopathic ventricular fibrillation (138). A single-pass type II membrane protein DPP6 can promote the cell surface expression of potassium channel KCND2 and regulate its activity and gating characteristics (139–141). Haplotype analysis in both familial and sporadic cases indicated the relevance of DPP6 with idiopathic ventricular fibrillation (133, 142). Furthermore, a DPP6 mutation was detected in idiopathic ventricular fibrillation patients, which disturbs the efflux of potassium ion (79). In addition, mutations of transcription factor IRX3, specifically expressed in His bundle, have also been reported to cause idiopathic ventricular fibrillation by down-regulating SCN5A and connexin-40 mRNA (80). Although several diagnostic approaches have been proposed and pathogenic mechanisms discussed, it is far from enough for studies of idiopathic ventricular fibrillation (143). This situation may change with the development of diagnostic techniques for structural heart disease and the discovery of potential pathogenic mechanisms.

#### Somatic Mutations

Besides the above germline mutations, i.e., mutations carried by all cells, the effect of somatic mutations, i.e., mutations carried by only some somatic cells, on the occurrence of ventricular arrhythmias is also being investigated. Idiopathic right ventricular outflow tract ventricular tachycardia (RVOT-VT) is the most common form of ventricular arrhythmias in patients without structural heart disease. GNAI2 (f200l) and A1AR (R296C) mutations were detected in biopsy samples collected from the origin of ventricular tachycardia in patients with adenosine insensitive RVOT tachycardia, but not in remote myocardium (18, 81). GNAI2 (F200L) increases intracellular cAMP concentrations and inhibited the inhibition of cAMP by adenosine, the effect of another mutation is unclear (81). Another study identified a cardiac somatic mutation (W234R) in GNAS (Gs-alpha, stimulatory G protein alpha-subunit) in endomyocardial biopsy samples from the origin of tachycardia in RVOT-VT patient, which was not detected in right ventricular apex biopsy samples. The mutation impairs GTP hydrolysis and increased basal intracellular cAMP levels, increasing the basal inward calcium current, and silico modeling show delayed afterdepolarizations and triggered activity (82).

### Polygenic Factors

In the process of screening disease-causing genes, we often tend to pay more attention to single-gene factors, emphasizing that a gene mutation corresponds to a clinical phenotype. However, molecular genetics is developing from single-gene research to multi-gene research. The same gene mutation can give rise to multiple distinct phenotypes. The SCN5A mutation detected in a Dutch family causes two phenotypes: LQTS and BrS (144). Mutations that have been reported to be pathogenic may appear no overt phenotype when they exist in other families or individuals, in the same pedigree, mostly display incomplete clinical penetrance (145, 146). It is more common that the same phenotype is caused by a mutation in different genes, in fact, it can also be caused by the accumulation of mutations in multiple different genes, and may affect the severity of the disease. For example, the interaction between KCND3 and SCN5A, Na_v_1.5 and K_v_4.3 channels regulates each other's functions through trafficking and gating mechanisms (147). When screening the LQTS susceptibility gene in a Caucasian family with syncope and slightly prolonged QT interval, it was found that there is R800L mutation in SCN5A and A261V mutation in SNTA1. Family members with both mutations have the strongest clinical Phenotype (19). In a GWAS study, two significant association signals were detected in Scn10a locus (rs10428132) and near the HEY2 gene (rs9388451) in patients with BrS, and an additional signal rs11708996 was identified in SCN5A, three common variations present an unexpectedly cumulative effect on disease susceptibility. The association signal of SCN5A-SCN10A indicate that genetic polymorphisms regulating cardiac conduction also affects the susceptibility to arrhythmia (20). In addition, the concept of genetic modifiers also indicates the cooperation between multiple genes. KCNQ1 mutation A341V carriers exhibit inconsistent QT intervals, and AKAP9, encoding a scaffolding protein, variants has been shown to alter the QTc interval of the population and the risk and severity of cardiac events (21, 148). A GWAS study conducted in China investigated the relationship between the NOS1AP gene rs12143842 variant and idiopathic ventricular tachycardia. The results showed that the minor T allele of the rs12143842 SNP was significantly associated with the reduced risk of idiopathic ventricular tachycardia in the Northern Chinese cohort, indicating that SNPs can affect the susceptibility to ventricular tachycardia (149). Consistently, a recent GWAS study with polygenic risk score analysis in BrS patients confirmed the association of mutation accumulation effects with traits (22).

## Epigenetic Factors of Ventricular Arrhythmias Without Structural Heart Disease

There are still a large number of cases of hereditary ventricular arrhythmias in which the causative gene has not been detected. For example, in BrS, approximately 20 pathogenic genes have been reported, but comprehensive single gene factors and polygenic factors still account for 60–80% of the causes unknown. In addition to fever, exercise, illness, and other factors, epigenetic factors have gradually attracted attention (150–152). Genetic research on diseases mainly focuses on the primary structure of DNA, i.e., when the DNA sequence changes, causing alterations in gene transcription and translation. Unlike genetics, epigenetics studies the molecules and mechanisms that maintain the state of selectable gene activity states without changing the DNA sequence and has mainly been studied in the fields of non-coding RNA expression, DNA methylation, histone modifications, genomic imprinting, and three-dimensional genome architecture (Table 1) (153).

### Non-coding RNA

Non-coding RNA refers to RNA that does not encode protein, which is transcribed from the genome and does not translate into protein, but performs biological functions at the RNA level, including microRNA, circular RNA, small nuclear RNA, long non-coding RNA, etc.

MicroRNA (miRNA) is a class of conservative non-coding small RNA, primarily involved in the regulation of post-transcriptional level that can cause target mRNA degradation or repress translation by specific base pairing with target mRNA, thereby affecting the expression of target mRNA. MiR-19b has been reported to be established as a potential candidate for human LQTS, with impaired repolarization of miR-19b-deficient zebrafish, significantly prolonged action potential, showing severe bradycardia and susceptibility to arrhythmias and cardiomyopathy. MiR-19b targets multiple ion channel-related genes. SCN1B acts as the β subunit of Na_v_1.5, directly regulated by miR-19b and is upregulated upon its loss, possibly leading to prolonged action potential duration by increasing late sodium current. Upon miR-19b reduction, both KCNE4 and KCNE1 are significantly upregulated, which may be due to the impaired cardiac repolarization caused by the inhibition of KCNQ1, resulting in reduced potassium currents, which leads to the prolongation of AP and bradycardia. In addition, the downregulation of the expression of KCNA4, KCND3, SCN12B and CACNA1C indirectly regulated by mir-19b was detected, in which KCNA4 and KCND3 mediate the I_to_. The notch decreased during early repolarization due to damaged I_to_, which may explain the increased potential observed in stage 1 of the miR-19b-deficient heart. However, the presence of I_to_ in zebrafish hearts remains controversial. Further, miR-19b reduction can significantly rescue the heterozygous zebrafishes with SQTS phenotype (23).

miRNA can exist stably in a variety of body fluids, known as circulating miRNA. Circulating miRNA levels are associated with multiple diseases, and the expression profile differences significantly between normal people and disease patients, which may be a new class of disease markers. In pediatric patients without organic heart disease, miR-133 plasma levels were increased in children with ventricular tachycardia as compared with healthy controls (24). Circulating miRNA studies in patients with idiopathic ventricular tachycardia and arrhythmogenic cardiomyopathy showed that the plasma levels of miRNA-320 in idiopathic ventricular tachycardia patients were significantly higher than those in ACM patients, which may help to distinguish idiopathic ventricular tachycardia and arrhythmogenic cardiomyopathy (154). MiRNA mostly combines with the UTR region of the coding gene to regulate gene expression, so the sequence of UTR regions is of great significance for controlling the expression of ion channel genes. In fact, there have been reported mutation detection in the SCN5A UTR region and biological predictions of the combined miRNA (25, 155). Variations were also detected in the UTR region of SCN1B, but the miRNAs that may bind to SCN1B were not described in detail (156). Scientists have also tried to screen the genetic mutations of miRNA in LQTS patients to explain the cause of LQTS, but the mutation sites found failed to explain the cause of the disease in the cohort (157).

It is known that KCNH2 encoding K_v_11.1, which is a subunit of rapid-acting inward rectifying potassium channel, actually KCNH2 intron 9 is splicing inefficient in human heart, only 1/3 of the precursor mRNA is processed into functional K_v_11.1a isomers and 2/3 into C-terminal truncated non-functional K_v_11.1a-USO isomers. The initial step of the splicing process involves the recognition of the 5' splice site by U1 small nuclear ribonucleoprotein. This recognition is mediated by complementary base pairing between the 5' splice site and U1 SnRNA (the RNA component of U1 small ribonucleoprotein) (158). A KCNH2 IVS9-2delA mutation was found in a large LQTS family that resulted in the switching of functional K_v_11.1a isoform to the non-functional K_v_11.1a-USO isoform (159). The extent of complementarity between the 5' splice site and U1 snRNA is an important determinant of splicing efficiency. Modifying the sequence of U1 snRNA can increase its complementarity to the 5' splice site of KCNH2 intron 9, significantly improve the splicing efficiency of the intron 9, increase the expression of the functional K_v_11.1a isoforms, and thereby increase the K_v_11.1 current (158).

### DNA Methylation

DNA methylation refers to the process where organisms transfer methyl groups to specific bases with S-adenosylmethionine as the methyl donor under DNA methyltransferase. DNA methylation is regulated at transcription levels and mainly plays an inhibitory role. The common SCN5A polymorphism H558R (rs1805124), a genetic modifier of BRS, can improve the electrocardiographic features and clinical phenotype of SCN5A mutation carriers by repairing abnormal channel gating kinetics and membrane trafficking and improving sodium channel activity in mutant channels (160–164). In fact, studies have shown that the SCN5A polymorphism H558R can reduce the methylation level of the SCN5A promoter, increase the expression level of SCN5A in the heart tissue, and prevent the occurrence of ventricular fibrillation (26). The G allele of H558R has been linked to QTc interval prolongation, which corresponds to its function (165, 166).

DNA methylation can also play a role in gene imprinting by forming differentially methylated regions (DMRs) on genomes of different parental origins to regulate gene expression (167). For example, there is a differential methylation region within the KCNQ1 locus (KvDMR1), which regulates multiple genes, including KCNQ1, long non-coding RNA KCNQ1OT1, and CDKN1C (27). Since both KCNQ1OT1 and KCNQ1 associated with LQTS are imprinted genes, this part is detailed in the genomic imprinting section.

### Histone Modifications

Nucleosome is the basic unit of chromatin composed of histones and DNA wrapped around histones. Modifications of nucleosomes control DNA packaging and regulate the entry of the transcription factor into the DNA. There are at least 15 functional histone modifications that can occur in cells, with histone methylation and demethylation, as well as acetylation and deacetylation, being the most commonly studied modifications in the heart (168).

Histone methylation can be associated with transcriptional activation or repression, while histone acetylation is usually associated with transcriptional activation (169, 170). The active trimethylation of histone H3 at lysine 4 (H3K4me3) mark plays a pivotal role in cell homeostasis in fully differentiated tissues. Decreased h3k4me3 reduces gene expression of KCHIP2, the β-subunit of the I_to_ channel, and attenuates I_to_ and sodium current, prolonging the action potential duration and resulting in increased I_Ca, L_ and enhanced cardiac contractile function (28, 29). KCHIP2 is heterogeneously expressed in the human and mouse ventricular walls, and although there have been no cases of association of KCHIP2 mutations with cardiac disease, functional studies have shown that the gene defect alters repolarization gradients, eliminates fast I_to_, and increases the susceptibility of murine ventricular cells to ventricular tachycardia (30).

HEY2 is a BrS susceptibility gene, which can not only affect the expression of SCN5A and the formation of the cardiac conduction system but also regulate the expression of transmural potassium channels such as Kcnip2 and KCND2, altering the peak and density of I_to_ and I_Na_ and affecting transmural electrophysiological gradients (20). Studies have shown that H3K4me3 and active H3K27ac (histone H3 acetylated lysine 27) can regulate the ventricular differential transcription of the HEY2 gene by binding to the promoter or enhancer of HEY2, affecting ventricular myocyte depolarization and repolarization (168).

### Genomic Imprinting

Genomic imprinting refers to the phenomenon that gene expression has parental selectivity in tissues or cells, and only a specific parental allele is expressed, and the other parental allele are not expressed or rarely expressed. Paternal genes are not expressed as paternal imprinting, and maternal imprinting is the same (171). The KCNQ1 gene, which is an imprinted gene, exists in clusters with other imprinted genes, called imprinted domains. Imprinted domains are regulated by shared regulatory elements (long non-coding RNA and DMR) (171). KCNQ1OT1 is a long non-coding RNA located in the KCNQ1 locus, with a promoter located within the KvDMR1. Affected by differential methylation of the promoter region this gene only expresses the paternal allele, while the maternal allele is silent (172). Unlike KCNQ1OT1, KCNQ1 is biallelic expression in adult tissues and fetal heart, although it shows maternal expression in other fetal tissues and the coding of paternal gene is repressed (27). The main reason is that KCNQ1OT1 can regulate the spatiotemporal expression of KCNQ1 by coordinating chromatin conformation changes and histone modifications. which makes LQTS caused by KCNQ1 mutation not show obvious maternal predisposition, but an autosomal dominant manner (173, 174). Even so, there are still reports that LQTS exhibits female predominance and transmission distortion, i.e., the LQTS allele is more maternally than paternally derived, especially in patients with LQTS due to KCNQ1 mutations, which may be associated with genomic imprinting (31, 32). A study investigating the methylation state of KCNQ1OT1 in a group of patients with symptomatic QTc prolongation found that the rs11023840 AA genotype of KCNQ1OT1 increased the methylation level of KCNQ1OT1 promoter, which was associated with prolonged QTc interval, supporting the role of differential methylation/imprinting of KCNQ1OT1 in symptomatic prolonged QTc risk (33). However, another study showed that different degrees of potassium channel dysfunction caused by variations in different parental origins may also lead to transmission distortion (175). Although inconclusive, there is no doubt that genetic imprinting plays an important role in the pathogenesis of LQTS.

Arsenic trioxide (As2O3, ATO) is a reagent for the treatment of acute promyelocytic leukemia with adverse effects, including the induction of LQTS. Studies have shown that ATO induces a decrease in the transcription level of long non-coding RNA KCNQ1OT1 in the KCNQ1 locus, while KCNQ1OT1 silencing can inhibit the expression of KCNQ1, thus prolonging action potential duration *in vitro* and causing LQTS *in vivo* (176). It has also been reported that miRNA is also involved in ATO-induced QT prolongation, with ATO-induced miR-133 and miR-1 dysregulation acting as the basis of As2O3-induced cardiac electrical disorder. In guinea pigs, ERG protein expression was inhibited by miR-133 while the expression of Kir2.1 channel protein was downregulated by miR-1, resulting in the decrease of rapidly activating delayed rectifier potassium current and inward rectifier potassium current, thereby inducing the prolongation of QT (177).

### Three-Dimensional Genome Architecture

With the development of chromosome conformation capture technologies and fluorescence *in situ* hybridization imaging technologies, the role of 3D genome architecture in gene regulation has gradually emerged (178, 179). The genomic DNA in the nucleus is folded into an ordered chromatin spatial structure that allows elements with a long linear distance can also interact, such as the loop structure formed between enhancer and promoter (180). Among them, CTCF and cohesin complexes assemble three-dimensional chromatin loops in the genome and play a crucial role in regulating the transcriptional patterns of genes (181, 182). CTCF and cohesins are abundantly enriched at topological domain boundaries and localize to CTCF sites, limiting the interaction of functional elements within topological domains (34, 183). Although the association of CTCF binding site variants with ventricular arrhythmias in non-structural heart disease has not been reported, however, CTCF knockout has been reported to cause disorder in the expression of RYR2, KCND2/KCNQ1/SCN5A/CACNB1 and other genes in the ventricle, leads to heart failure, suggesting an association with ventricular arrhythmias (35, 36). Besides, DNA sequence changes that affect the formation of the normal 3D structure of the genome have also been confirmed to be involved in the occurrence and development of the disease (184). Studies have shown that the enhancer (ENHA) in SCN10A interacts with SCN5A promoter, which is necessary for the expression of SCN5A *in vivo* (185). The major allele G of the common variant locus rs6801957 in SCN10A located within this enhancer region establishes a conserved T-box transcription factor binding site to promote enhancer activity, while the risk allele significantly reduces the expression of SCN5A (186). This largely explains the association of this variant site with QRS prolongation in the GWAS study and common variants in SCN10A with BrS (20, 187). In turn, the results of GWAS can also guide the discovery of functional element regions within the loci (188, 189). The analysis of common variants associated with LQTS in the KCNH2 locus identified a conserved cardiac cis-acting element that acts as enhancer and regulates KCNH2 expression through physical proximity to the KCNH2 promoter (37). In fact, GWAS studies have identified a large number of SNPs associated with heart disease, however, the mechanisms of most of the SNPs involved in the occurrence of the disease have not been revealed (190). Marking of regulatory element regions such as enhancers of known disease-causing genes can characterize some variants of unknown significance and provide a theoretical basis for deciphering genetic variation (191). In conclusion, studies of 3D genome architecture provide a new perspective for us to understand the potential mechanism between these SNPs and diseases, as well as a better understanding of the pathogenic mechanism of diseases.

## Summary

This article summarizes the genetic and epigenetic factors of ventricular arrhythmia in patients without structural heart disease and introduces the genes that may cause ventricular arrhythmias and their possible pathogenic mechanisms. However, there are still a large number of cases of ventricular arrhythmias without structural heart disease whose causes have not been clarified. Further research and exploration are needed, and the development of high-throughput patch clamping and other related technologies will undoubtedly play an important role. In addition, emerging disciplines such as optogenetics are also developing steadily, which will drive us to have a deeper understanding of the pathogenesis of ventricular arrhythmias.

## Author Contributions

XT: conceptualization, writing, and supervision. MW: data curation and writing. Both authors contributed to the article and approved the submitted version.

## Conflict of Interest

The authors declare that the research was conducted in the absence of any commercial or financial relationships that could be construed as a potential conflict of interest.

## Publisher's Note

All claims expressed in this article are solely those of the authors and do not necessarily represent those of their affiliated organizations, or those of the publisher, the editors and the reviewers. Any product that may be evaluated in this article, or claim that may be made by its manufacturer, is not guaranteed or endorsed by the publisher.

## References

[1] Ortmans S Daval C Aguilar M Compagno P Cadrin-Tourigny J Dyrda K. Pharmacotherapy in inherited and acquired ventricular arrhythmia in structurally normal adult hearts. Expert Opin Pharmacother. (2019) 20:2101–14. 10.1080/14656566.2019.1669561 31566420

[2] Prystowsky EN Padanilam BJ Joshi S Fogel RI. Ventricular arrhythmias in the absence of structural heart disease. J Am Coll Cardiol. (2012) 59:1733–44. 10.1016/j.jacc.2012.01.036 22575310

[3] Killu AM Stevenson WG. Ventricular tachycardia in the absence of structural heart disease. Heart. (2019) 105:645–56. 10.1136/heartjnl-2017-311590 30541758

[4] Nademanee K Veerakul G Nogami A Lou Q Hocini M Coronel R. Mechanism of the effects of sodium channel blockade on the arrhythmogenic substrate of Brugada syndrome. Heart Rhythm. (2022) 19:407–16. 10.1016/j.hrthm.2021.10.031 34742919

[5] Nademanee K Tei C. Two faces of brugada syndrome: electrical and structural diseases. JACC Clin Electrophysiol. (2020) 6:1364–6. 10.1016/j.jacep.2020.07.006 33121664

[6] Liu J Laksman Z Backx PH. The electrophysiological development of cardiomyocytes. Adv Drug Deliv Rev. (2016) 96:253–73. 10.1016/j.addr.2015.12.023 26788696

[7] Rivaud MR Delmar M Remme CA. Heritable arrhythmia syndromes associated with abnormal cardiac sodium channel function: ionic and non-ionic mechanisms. Cardiovasc Res. (2020) 116:1557–70. 10.1093/cvr/cvaa082 32251506 PMC7341171

[8] Jeyaraj D Ashwath M Rosenbaum DS. Pathophysiology and clinical implications of cardiac memory. Pacing Clin Electrophysiol. (2010) 33:346–52. 10.1111/j.1540-8159.2009.02630.x 20025710 PMC2865579

[9] Eisner DA Caldwell JL Kistamas K Trafford AW. Calcium and excitation-contraction coupling in the heart. Circ Res. (2017) 121:181–95. 10.1161/CIRCRESAHA.117.310230 28684623 PMC5497788

[10] Monteiro LM Vasques-Novoa F Ferreira L Pinto-do-O P Nascimento DS. Restoring heart function and electrical integrity: closing the circuit. NPJ Regen Med. (2017) 2:9. 10.1038/s41536-017-0015-2 29302345 PMC5665620

[11] Brandao KO Tabel VA Atsma DE Mummery CL Davis RP. Human pluripotent stem cell models of cardiac disease: from mechanisms to therapies. Dis Model Mech. (2017) 10:1039–59. 10.1242/dmm.030320 28883014 PMC5611968

[12] Blaustein MP Lederer WJ. Sodium/calcium exchange: its physiological implications. Physiol Rev. (1999) 79:763–854. 10.1152/physrev.1999.79.3.763 10390518

[13] Fozzard HA. Afterdepolarizations and triggered activity. Basic Res Cardiol. (1992) 87 (Suppl. 2):105–13. 10.1007/978-3-642-72477-0_10 1299205

[14] DiFrancesco D. A brief history of pacemaking. Front Physiol. (2019) 10:1599. 10.3389/fphys.2019.01599 32038284 PMC6987461

[15] Wilde A Amin AS. Clinical spectrum of SCN5A mutations: long QT syndrome, brugada syndrome, and cardiomyopathy. JACC Clin Electrophysiol. (2018) 4:569–79. 10.1016/j.jacep.2018.03.006 29798782

[16] Kapplinger JD Tester DJ Salisbury BA Carr JL Harris-Kerr C Pollevick GD. Spectrum and prevalence of mutations from the first 2,500 consecutive unrelated patients referred for the FAMILION long QT syndrome genetic test. Heart Rhythm. (2009) 6:1297–303. 10.1016/j.hrthm.2009.05.021 19716085 PMC3049907

[17] Medeiros-Domingo A Bhuiyan ZA Tester DJ Hofman N Bikker H van Tintelen JP. The RYR2-encoded ryanodine receptor/calcium release channel in patients diagnosed previously with either catecholaminergic polymorphic ventricular tachycardia or genotype negative, exercise-induced long QT syndrome: a comprehensive open reading frame mutational analysis. J Am Coll Cardiol. (2009) 54:2065–74. 10.1016/j.jacc.2009.08.022 19926015 PMC2880864

[18] Lerman BB Dong B Stein KM Markowitz SM Linden J Catanzaro DF. Right ventricular outflow tract tachycardia due to a somatic cell mutation in G protein subunitalphai2. J Clin Invest. (1998) 101:2862–8. 10.1172/JCI1582 9637720 PMC508877

[19] Hu RM Tan BH Orland KM Valdivia CR Peterson A Pu J. Digenic inheritance novel mutations in SCN5a and SNTA1 increase late I(Na) contributing to LQT syndrome. Am J Physiol Heart Circ Physiol. (2013) 304:H994–1001. 10.1152/ajpheart.00705.2012 23376825 PMC3625899

[20] Bezzina CR Barc J Mizusawa Y Remme CA Gourraud JB Simonet F. Common variants at SCN5A-SCN10A and HEY2 are associated with Brugada syndrome, a rare disease with high risk of sudden cardiac death. Nat Genet. (2013) 45:1044–9. 10.1038/ng.2712 23872634 PMC3869788

[21] de Villiers CP van der Merwe L Crotti L Goosen A George AJ Schwartz PJ. AKAP9 is a genetic modifier of congenital long-QT syndrome type 1. Circ Cardiovasc Genet. (2014) 7:599–606. 10.1161/CIRCGENETICS.113.000580 25087618 PMC4270884

[22] Barc J Tadros R Glinge C Chiang DY Jouni M Simonet F. Genome-wide association analyses identify new Brugada syndrome risk loci and highlight a new mechanism of sodium channel regulation in disease susceptibility. Nat Genet. (2022) 54:232–9. 10.1038/s41588-021-01007-6 35210625 PMC9376964

[23] Benz A Kossack M Auth D Seyler C Zitron E Juergensen L. MiR-19b regulates ventricular action potential duration in zebrafish. Sci Rep. (2016) 6:36033. 10.1038/srep36033 27805004 PMC5090966

[24] Sun L Sun S Zeng S Li Y Pan W Zhang Z. Expression of circulating microRNA-1 and microRNA-133 in pediatric patients with tachycardia. Mol Med Rep. (2015) 11:4039–46. 10.3892/mmr.2015.3246 25625292 PMC4394928

[25] Daimi H Khelil AH Neji A Ben HK Maaoui S Aranega A. Role of SCN5A coding and non-coding sequences in Brugada syndrome onset: what's behind the scenes? Biomed J. (2019) 42:252–60. 10.1016/j.bj.2019.03.003 31627867 PMC6818142

[26] Matsumura H Nakano Y Ochi H Onohara Y Sairaku A Tokuyama T. H558R, a common SCN5A polymorphism, modifies the clinical phenotype of Brugada syndrome by modulating DNA methylation of SCN5A promoters. J Biomed Sci. (2017) 24:91. 10.1186/s12929-017-0397-x 29202755 PMC5713129

[27] Mancini-DiNardo D Steele SJ Ingram RS Tilghman SM. A differentially methylated region within the gene Kcnq1 functions as an imprinted promoter and silencer. Hum Mol Genet. (2003) 12:283–94. 10.1093/hmg/ddg024 12554682

[28] Rosati B Pan Z Lypen S Wang HS Cohen I Dixon JE. Regulation of KChIP2 potassium channel beta subunit gene expression underlies the gradient of transient outward current in canine and human ventricle. J Physiol. (2001) 533:119–25. 10.1111/j.1469-7793.2001.0119b.x 11351020 PMC2278594

[29] Stein AB Jones TA Herron TJ Patel SR Day SM Noujaim SF. Loss of H3K4 methylation destabilizes gene expression patterns and physiological functions in adult murine cardiomyocytes. J Clin Invest. (2011) 121:2641–50. 10.1172/JCI44641 21646717 PMC3223825

[30] Kuo HC Cheng CF Clark RB Lin JJ Lin JL Hoshijima M. A defect in the Kv channel-interacting protein 2 (KChIP2) gene leads to a complete loss of I(to) and confers susceptibility to ventricular tachycardia. Cell. (2001) 107:801–13. 10.1016/S0092-8674(01)00588-8 11747815

[31] Imboden M Swan H Denjoy I Van Langen IM Latinen-Forsblom PJ Napolitano C. Female predominance and transmission distortion in the long-QT syndrome. N Engl J Med. (2006) 355:2744–51. 10.1056/NEJMoa042786 17192539

[32] Lorca R Junco-Vicente A Martin-Fernandez M Pascual I Aparicio A Barja N. Clinical implications and gender differences of KCNQ1 p.Gly168Arg pathogenic variant in long QT syndrome. J Clin Med. (2020) 9:3846. 10.3390/jcm9123846 33256261 PMC7760054

[33] Coto E Calvo D Reguero JR Moris C Rubin JM Diaz-Corte C. Differential methylation of lncRNA KCNQ1OT1 promoter polymorphism was associated with symptomatic cardiac long QT. Epigenomics-Uk. (2017) 9:1049–57. 10.2217/epi-2017-0024 28749187

[34] Dixon JR Selvaraj S Yue F Kim A Li Y Shen Y. Topological domains in mammalian genomes identified by analysis of chromatin interactions. Nature. (2012) 485:376–80. 10.1038/nature11082 22495300 PMC3356448

[35] Lee DP Tan W Anene-Nzelu CG Lee C Li PY Luu T. Robust CTCF-Based chromatin architecture underpins epigenetic changes in the heart failure stress-gene response. Circulation. (2019) 139:1937–56. 10.1161/CIRCULATIONAHA.118.036726 30717603

[36] Rosa-Garrido M Chapski DJ Schmitt AD Kimball TH Karbassi E Monte E. High-Resolution mapping of chromatin conformation in cardiac myocytes reveals structural remodeling of the epigenome in heart failure. Circulation. (2017) 136:1613–25. 10.1161/CIRCULATIONAHA.117.029430 28802249 PMC5648689

[37] van den Boogaard M van Weerd JH Bawazeer AC Hooijkaas IB van de Werken H Tessadori F. Identification and characterization of a transcribed distal enhancer involved in cardiac kcnh2 regulation. Cell Rep. (2019) 28:2704–14. 10.1016/j.celrep.2019.08.007 31484079

[38] Watanabe H Koopmann TT Le Scouarnec S Yang T Ingram CR Schott JJ. Sodium channel beta1 subunit mutations associated with Brugada syndrome and cardiac conduction disease in humans. J Clin Invest. (2008) 118:2260–8. 10.1172/JCI33891 18464934 PMC2373423

[39] Riuro H Beltran-Alvarez P Tarradas A Selga E Campuzano O Verges M. A missense mutation in the sodium channel beta2 subunit reveals SCN2B as a new candidate gene for Brugada syndrome. Hum Mutat. (2013) 34:961–6. 10.1002/humu.22328 23559163

[40] Dulsat G Palomeras S Cortada E Riuro H Brugada R Verges M. Trafficking and localisation to the plasma membrane of Nav 1.5 promoted by the beta2 subunit is defective due to a beta2 mutation associated with Brugada syndrome. Biol Cell. (2017) 109:273–91. 10.1111/boc.201600085 28597987

[41] Ishikawa T Takahashi N Ohno S Sakurada H Nakamura K On On YK. Novel SCN3B mutation associated with brugada syndrome affects intracellular trafficking and function of Nav1.5. Circ J. (2013) 77:959–67. 10.1253/circj.CJ-12-0995 23257389

[42] Medeiros-Domingo A Kaku T Tester DJ Iturralde-Torres P Itty A Ye B. SCN4B-encoded sodium channel beta4 subunit in congenital long-QT syndrome. Circulation. (2007) 116:134–42. 10.1161/CIRCULATIONAHA.106.659086 17592081 PMC3332546

[43] London B Michalec M Mehdi H Zhu X Kerchner L Sanyal S. Mutation in glycerol-3-phosphate dehydrogenase 1 like gene (GPD1-L) decreases cardiac Na+ current and causes inherited arrhythmias. Circulation. (2007) 116:2260–8. 10.1161/CIRCULATIONAHA.107.703330 17967977 PMC3150966

[44] Kattygnarath D Maugenre S Neyroud N Balse E Ichai C Denjoy I. MOG1: a new susceptibility gene for Brugada syndrome. Circ Cardiovasc Genet. (2011) 4:261–8. 10.1161/CIRCGENETICS.110.959130 21447824

[45] Yu G Liu Y Qin J Wang Z Hu Y Wang F. Mechanistic insights into the interaction of the MOG1 protein with the cardiac sodium channel Nav1.5 clarify the molecular basis of Brugada syndrome. J Biol Chem. (2018) 293:18207–17. 10.1074/jbc.RA118.003997 30282806 PMC6254340

[46] Hu D Barajas-Martinez H Pfeiffer R Dezi F Pfeiffer J Buch T. Mutations in SCN10A are responsible for a large fraction of cases of Brugada syndrome. J Am Coll Cardiol. (2014) 64:66–79. 10.1016/j.jacc.2014.04.032 24998131 PMC4116276

[47] Wu X Jiang X Li H Tian P Zhou Z Shi S. Expression of HCN4 protein in ventricular outflow tract of rabbit with idiopathic ventricular tachycardia. Zhonghua Yi Xue Za Zhi. (2015) 95:3620–4. 26813378

[48] Li X Li Z Wang D Wang DW Wang Y. A novel gain-of-function KCND3 variant associated with Brugada syndrome. Cardiology. (2020) 145:623–32. 10.1159/000508033 32818936

[49] Delpon E Cordeiro JM Nunez L Thomsen PE Guerchicoff A Pollevick GD. Functional effects of KCNE3 mutation and its role in the development of Brugada syndrome. Circ Arrhythm Electrophysiol. (2008) 1:209–18. 10.1161/CIRCEP.107.748103 19122847 PMC2585750

[50] Ohno S Zankov DP Ding WG Itoh H Makiyama T Doi T. KCNE5 (KCNE1L) variants are novel modulators of Brugada syndrome and idiopathic ventricular fibrillation. Circ Arrhythm Electrophysiol. (2011) 4:352–61. 10.1161/CIRCEP.110.959619 21493962

[51] Wang Q Curran ME Splawski I Burn TC Millholland JM VanRaay TJ. Positional cloning of a novel potassium channel gene: KVLQT1 mutations cause cardiac arrhythmias. Nat Genet. (1996) 12:17–23. 10.1038/ng0196-17 8528244

[52] Bellocq C van Ginneken AC Bezzina CR Alders M Escande D Mannens MM. Mutation in the KCNQ1 gene leading to the short QT-interval syndrome. Circulation. (2004) 109:2394–7. 10.1161/01.CIR.0000130409.72142.FE 15159330

[53] Garmany R Giudicessi JR Ye D Zhou W Tester DJ Ackerman MJ. Clinical and functional reappraisal of alleged type 5 long QT syndrome: causative genetic variants in the KCNE1-encoded minK beta-subunit. Heart Rhythm. (2020) 17:937–44. 10.1016/j.hrthm.2020.02.003 32058015

[54] Ono M Burgess DE Schroder EA Elayi CS Anderson CL January CT. Long QT syndrome type 2: emerging strategies for correcting class 2 KCNH2 (hERG) mutations and identifying new patients. Biomolecules. (2020) 10:1144. 10.3390/biom10081144 32759882 PMC7464307

[55] Brugada R Hong K Dumaine R Cordeiro J Gaita F Borggrefe M. Sudden death associated with short-QT syndrome linked to mutations in HERG. Circulation. (2004) 109:30–5. 10.1161/01.CIR.0000109482.92774.3A 14676148

[56] Isbrandt D Friederich P Solth A Haverkamp W Ebneth A Borggrefe M. Identification and functional characterization of a novel KCNE2 (MiRP1) mutation that alters HERG channel kinetics. J Mol Med. (2002) 80:524–32. 10.1007/s00109-002-0364-0 12185453

[57] Priori SG Pandit SV Rivolta I Berenfeld O Ronchetti E Dhamoon A. A novel form of short QT syndrome (SQT3) is caused by a mutation in the KCNJ2 gene. Circ Res. (2005) 96:800–7. 10.1161/01.RES.0000162101.76263.8c 15761194

[58] Fodstad H Swan H Auberson M Gautschi I Loffing J Schild L. Loss-of-function mutations of the K(+) channel gene KCNJ2 constitute a rare cause of long QT syndrome. J Mol Cell Cardiol. (2004) 37:593–602. 10.1016/j.yjmcc.2004.05.011 15276028

[59] Wang F Liu J Hong L Liang B Graff C Yang Y. The phenotype characteristics of type 13 long QT syndrome with mutation in KCNJ5 (Kir3.4-G387R). Heart Rhythm. (2013) 10:1500–6. 10.1016/j.hrthm.2013.07.022 23872692

[60] Barajas-Martinez H Hu D Ferrer T Onetti CG Wu Y Burashnikov E. Molecular genetic and functional association of Brugada and early repolarization syndromes with S422L missense mutation in KCNJ8. Heart Rhythm. (2012) 9:548–55. 10.1016/j.hrthm.2011.10.035 22056721 PMC3288170

[61] Hu D Barajas-Martinez H Terzic A Park S Pfeiffer R Burashnikov E. ABCC9 is a novel Brugada and early repolarization syndrome susceptibility gene. Int J Cardiol. (2014) 171:431–42. 10.1016/j.ijcard.2013.12.084 24439875 PMC3947869

[62] Wemhoner K Friedrich C Stallmeyer B Coffey AJ Grace A Zumhagen S. Gain-of-function mutations in the calcium channel CACNA1C (Cav1.2) cause non-syndromic long-QT but not Timothy syndrome. J Mol Cell Cardiol. (2015) 80:186–95. 10.1016/j.yjmcc.2015.01.002 25633834

[63] Di Mauro V Ceriotti P Lodola F Salvarani N Modica J Bang ML. Peptide-Based targeting of the L-type calcium channel corrects the loss-of-function phenotype of two novel mutations of the CACNA1 gene associated with Brugada syndrome. Front Physiol. (2020) 11:616819. 10.3389/fphys.2020.616819 33488405 PMC7821386

[64] Burashnikov E Pfeiffer R Barajas-Martinez H Delpon E Hu D Desai M. Mutations in the cardiac L-type calcium channel associated with inherited J-wave syndromes and sudden cardiac death. Heart Rhythm. (2010) 7:1872–82. 10.1016/j.hrthm.2010.08.026 20817017 PMC2999985

[65] Wleklinski MJ Kannankeril PJ Knollmann BC. Molecular and tissue mechanisms of catecholaminergic polymorphic ventricular tachycardia. J Physiol. (2020) 598:2817–34. 10.1113/JP276757 32115705 PMC7699301

[66] Zhong X Guo W Wei J Tang Y Liu Y Zhang JZ. Identification of loss-of-function RyR2 mutations associated with idiopathic ventricular fibrillation and sudden death. Biosci Rep. (2021) 41: BSR20210209. 10.1042/BSR20210209 33825858 PMC8062958

[67] Wang Q Michalak M. Calsequestrin. Structure, function, and evolution. Cell Calcium. (2020) 90:102242. 10.1016/j.ceca.2020.102242 32574906

[68] Roux-Buisson N Cacheux M Fourest-Lieuvin A Fauconnier J Brocard J Denjoy I. Absence of triadin, a protein of the calcium release complex, is responsible for cardiac arrhythmia with sudden death in human. Hum Mol Genet. (2012) 21:2759–67. 10.1093/hmg/dds104 22422768 PMC3363337

[69] Altmann HM Tester DJ Will ML Middha S Evans JM Eckloff BW. Homozygous/Compound heterozygous triadin mutations associated with autosomal-recessive long-QT syndrome and pediatric sudden cardiac arrest: elucidation of the triadin knockout syndrome. Circulation. (2015) 131:2051–60. 10.1161/CIRCULATIONAHA.115.015397 25922419

[70] Kotta MC Sala L Ghidoni A Badone B Ronchi C Parati G. Calmodulinopathy: a novel, life-threatening clinical entity affecting the young. Front Cardiovasc Med. (2018) 5:175. 10.3389/fcvm.2018.00175 30574507 PMC6291462

[71] Wu G Ai T Kim JJ Mohapatra B Xi Y Li Z. Alpha-1-syntrophin mutation and the long-QT syndrome: a disease of sodium channel disruption. Circ Arrhythm Electrophysiol. (2008) 1:193–201. 10.1161/CIRCEP.108.769224 19684871 PMC2726717

[72] Ishikawa T Sato A Marcou CA Tester DJ Ackerman MJ Crotti L. A novel disease gene for Brugada syndrome: sarcolemmal membrane-associated protein gene mutations impair intracellular trafficking of hNav1.5. Circ Arrhythm Electrophysiol. (2012) 5:1098–107. 10.1161/CIRCEP.111.969972 23064965

[73] Persampieri S Pilato CA Sommariva E Maione AS Stadiotti I Ranalletta A. Clinical and molecular data define a diagnosis of arrhythmogenic cardiomyopathy in a carrier of a Brugada-syndrome-associated PKP2 mutation. Genes. (2020) 11:571. 10.3390/genes11050571 32443836 PMC7288341

[74] Mohler PJ Schott JJ Gramolini AO Dilly KW Guatimosim S DuBell WH. Ankyrin-B mutation causes type 4 long-QT cardiac arrhythmia and sudden cardiac death. Nature. (2003) 421:634–9. 10.1038/nature01335 12571597

[75] Zaza A Grandi E. Mechanisms of Cav3-associated arrhythmia: protein or microdomain dysfunction? Int J Cardiol. (2020) 320:97–9. 10.1016/j.ijcard.2020.06.051 32634502 PMC7577955

[76] Devalla HD Gelinas R Aburawi EH Beqqali A Goyette P Freund C. TECRL, a new life-threatening inherited arrhythmia gene associated with overlapping clinical features of both LQTS and CPVT. Embo Mol Med. (2016) 8:1390–408. 10.15252/emmm.201505719 27861123 PMC5167130

[77] Thorsen K Dam VS Kjaer-Sorensen K Pedersen LN Skeberdis VA Jurevicius J. Loss-of-activity-mutation in the cardiac chloride-bicarbonate exchanger AE3 causes short QT syndrome. Nat Commun. (2017) 8:1696. 10.1038/s41467-017-01630-0 29167417 PMC5700076

[78] Amarouch MY El HJ. Inherited cardiac arrhythmia syndromes: focus on molecular mechanisms underlying TRPM4 channelopathies. Cardiovasc Ther. (2020) 2020:6615038. 10.1155/2020/6615038 33381229 PMC7759408

[79] Ding DB Fan LL Xiao Z Huang H Chen YQ Guo S. A novel mutation of dipeptidyl aminopeptidase-like protein-6 in a family with suspicious idiopathic ventricular fibrillation. QJM. (2018) 111:373–7. 10.1093/qjmed/hcy033 29474731

[80] Koizumi A Sasano T Kimura W Miyamoto Y Aiba T Ishikawa T. Genetic defects in a His-Purkinje system transcription factor, IRX3, cause lethal cardiac arrhythmias. Eur Heart J. (2016) 37:1469–75. 10.1093/eurheartj/ehv449 26429810 PMC4914882

[81] Cheung JW Ip JE Yarlagadda RK Liu CF Thomas G Xu L. Adenosine-insensitive right ventricular tachycardia: novel variant of idiopathic outflow tract tachycardia. Heart Rhythm. (2014) 11:1770–8. 10.1016/j.hrthm.2014.06.014 24931634

[82] Ip JE Xu L Dai J Steegborn C Jaffre F Evans T. Constitutively activating GNAS somatic mutation in right ventricular outflow tract tachycardia. Circ Arrhythm Electrophysiol. (2021) 14:e10082. 10.1161/CIRCEP.121.010082 34587755 PMC8569928

[83] Han D Tan H Sun C Li G. Dysfunctional Nav1.5 channels due to SCN5A mutations. Exp Biol Med. (2018) 243:852–63. 10.1177/1535370218777972 29806494 PMC6022915

[84] Valdivia CR Ueda K Ackerman MJ Makielski JC. GPD1L links redox state to cardiac excitability by PKC-dependent phosphorylation of the sodium channel SCN5A. Am J Physiol Heart Circ Physiol. (2009) 297:H1446–52. 10.1152/ajpheart.00513.2009 19666841 PMC2770765

[85] Biel S Aquila M Hertel B Berthold A Neumann T DiFrancesco D. Mutation in S6 domain of HCN4 channel in patient with suspected Brugada syndrome modifies channel function. Pflugers Arch. (2016) 468:1663–71. 10.1007/s00424-016-1870-1 27553229

[86] Ueda K Hirano Y Higashiuesato Y Aizawa Y Hayashi T Inagaki N. Role of HCN4 channel in preventing ventricular arrhythmia. J Hum Genet. (2009) 54:115–21. 10.1038/jhg.2008.16 19165230

[87] Niwa N Nerbonne JM. Molecular determinants of cardiac transient outward potassium current (I(to)) expression and regulation. J Mol Cell Cardiol. (2010) 48:12–25. 10.1016/j.yjmcc.2009.07.013 19619557 PMC2813406

[88] Amberg GC Koh SD Imaizumi Y Ohya S Sanders KM. A-type potassium currents in smooth muscle. Am J Physiol Cell Physiol. (2003) 284:C583–95. 10.1152/ajpcell.00301.2002 12556357

[89] Wu W Sanguinetti MC. Molecular basis of cardiac delayed rectifier potassium channel function and pharmacology. Card Electrophysiol Clin. (2016) 8:275–84. 10.1016/j.ccep.2016.01.002 27261821 PMC4894371

[90] Sanguinetti MC Curran ME Zou A Shen J Spector PS Atkinson DL. Coassembly of K(V)LQT1 and minK (IsK) proteins to form cardiac I(Ks) potassium channel. Nature. (1996) 384:80–3. 10.1038/384080a0 8900283

[91] Barhanin J Lesage F Guillemare E Fink M Lazdunski M Romey G. K(V)LQT1 and lsK (minK) proteins associate to form the I(Ks) cardiac potassium current. Nature. (1996) 384:78–80. 10.1038/384078a0 8900282

[92] Wallace E Howard L Liu M O'Brien T Ward D Shen S. Long QT syndrome: genetics and future perspective. Pediatr Cardiol. (2019) 40:1419–30. 10.1007/s00246-019-02151-x 31440766 PMC6785594

[93] Dvir M Strulovich R Sachyani D Ben-Tal CI Haitin Y Dessauer C. Long QT mutations at the interface between KCNQ1 helix C and KCNE1 disrupt I(KS) regulation by PKA and PIP(2). J Cell Sci. (2014) 127:3943–55. 10.1242/jcs.147033 25037568 PMC6519428

[94] Wu ZJ Huang Y Fu YC Zhao XJ Zhu C Zhang Y. Characterization of a Chinese KCNQ1 mutation (R259H) that shortens repolarization and causes short QT syndrome 2. J Geriatr Cardiol. (2015) 12:394–401. 10.11909/j.issn.1671-5411.2015.04.002 26346102 PMC4554793

[95] Roberts JD Asaki SY Mazzanti A Bos JM Tuleta I Muir AR. An international multicenter evaluation of type 5 long QT syndrome: a low penetrant primary arrhythmic condition. Circulation. (2020) 141:429–39. 10.1161/CIRCULATIONAHA.119.043114 31941373 PMC7035205

[96] Liu L Tian J Lu C Chen X Fu Y Xu B. Electrophysiological characteristics of the LQT2 syndrome mutation KCNH2-G572S and regulation by accessory protein KCNE2. Front Physiol. (2016) 7:650. 10.3389/fphys.2016.00650 28082916 PMC5187237

[97] Adeniran I El HA Hancox JC Zhang H. Proarrhythmia in KCNJ2-linked short QT syndrome: insights from modelling. Cardiovasc Res. (2012) 94:66–76. 10.1093/cvr/cvs082 22308236

[98] Plaster NM Tawil R Tristani-Firouzi M Canun S Bendahhou S Tsunoda A. Mutations in Kir2.1 cause the developmental and episodic electrical phenotypes of Andersen's syndrome. Cell. (2001) 105:511–9. 10.1016/S0092-8674(01)00342-7 11371347

[99] Hibino H Inanobe A Furutani K Murakami S Findlay I Kurachi Y. Inwardly rectifying potassium channels: their structure, function, and physiological roles. Physiol Rev. (2010) 90:291–366. 10.1152/physrev.00021.2009 20086079

[100] Medeiros-Domingo A Tan BH Crotti L Tester DJ Eckhardt L Cuoretti A. Gain-of-function mutation S422L in the KCNJ8-encoded cardiac K(ATP) channel Kir6.1 as a pathogenic substrate for J-wave syndromes. Heart Rhythm. (2010) 7:1466–71. 10.1016/j.hrthm.2010.06.016 20558321 PMC3049900

[101] Lariccia V Piccirillo S Preziuso A Amoroso S Magi S. Cracking the code of sodium/calcium exchanger (NCX) gating: old and new complexities surfacing from the deep web of secondary regulations. Cell Calcium. (2020) 87:102169. 10.1016/j.ceca.2020.102169 32070925

[102] Catterall WA. Signaling complexes of voltage-gated sodium and calcium channels. Neurosci Lett. (2010) 486:107–16. 10.1016/j.neulet.2010.08.085 20816922 PMC3433163

[103] Zhang Q Chen J Qin Y Wang J Zhou L. Mutations in voltage-gated L-type calcium channel: implications in cardiac arrhythmia. Channels. (2018) 12:201–18. 10.1080/19336950.2018.1499368 30027834 PMC6104696

[104] Fukuyama M Wang Q Kato K Ohno S Ding WG Toyoda F. Long QT syndrome type 8: novel CACNA1C mutations causing QT prolongation and variant phenotypes. Europace. (2014) 16:1828–37. 10.1093/europace/euu063 24728418

[105] Splawski I Timothy KW Sharpe LM Decher N Kumar P Bloise R. Ca(V)1.2 calcium channel dysfunction causes a multisystem disorder including arrhythmia and autism. Cell. (2004) 119:19–31. 10.1016/j.cell.2004.09.011 15454078

[106] Splawski I Timothy KW Decher N Kumar P Sachse FB Beggs AH. Severe arrhythmia disorder caused by cardiac L-type calcium channel mutations. Proc Natl Acad Sci USA. (2005) 102:8089–96, 8086–8. 10.1073/pnas.0502506102 15863612 PMC1149428

[107] Colson C Mittre H Busson A Leenhardt A Denjoy I Fressard V. Unusual clinical description of adult with Timothy syndrome, carrier of a new heterozygote mutation of CACNA1C. Eur J Med Genet. (2019) 62:103648. 10.1016/j.ejmg.2019.04.005 30998997

[108] Gillis J Burashnikov E Antzelevitch C Blaser S Gross G Turner L. Long QT, syndactyly, joint contractures, stroke and novel CACNA1C mutation: expanding the spectrum of Timothy syndrome. Am J Med Genet A. (2012) 158A:182–7. 10.1002/ajmg.a.34355 22106044 PMC3319791

[109] Boczek NJ Best JM Tester DJ Giudicessi JR Middha S Evans JM. Exome sequencing and systems biology converge to identify novel mutations in the L-type calcium channel, CACNA1C, linked to autosomal dominant long QT syndrome. Circ Cardiovasc Genet. (2013) 6:279–89. 10.1161/CIRCGENETICS.113.000138 23677916 PMC3760222

[110] Antzelevitch C Pollevick GD Cordeiro JM Casis O Sanguinetti MC Aizawa Y. Loss-of-function mutations in the cardiac calcium channel underlie a new clinical entity characterized by ST-segment elevation, short QT intervals, and sudden cardiac death. Circulation. (2007) 115:442–9. 10.1161/CIRCULATIONAHA.106.668392 17224476 PMC1952683

[111] Cordeiro JM Marieb M Pfeiffer R Calloe K Burashnikov E Antzelevitch C. Accelerated inactivation of the L-type calcium current due to a mutation in CACNB2b underlies Brugada syndrome. J Mol Cell Cardiol. (2009) 46:695–703. 10.1016/j.yjmcc.2009.01.014 19358333 PMC2668128

[112] Templin C Ghadri JR Rougier JS Baumer A Kaplan V Albesa M. Identification of a novel loss-of-function calcium channel gene mutation in short QT syndrome (SQTS6). Eur Heart J. (2011) 32:1077–88. 10.1093/eurheartj/ehr076 21383000 PMC3086900

[113] Liu W Deng J Wang G Zhang C Luo X Yan D. KCNE2 modulates cardiac L-type Ca(2+) channel. J Mol Cell Cardiol. (2014) 72:208–18. 10.1016/j.yjmcc.2014.03.013 24681347

[114] Baltogiannis GG Lysitsas DN di Giovanni G Ciconte G Sieira J Conte G. CPVT: Arrhythmogenesis, therapeutic management, and future perspectives. A brief review of the literature. Front Cardiovasc Med. (2019) 6:92. 10.3389/fcvm.2019.00092 31380394 PMC6644488

[115] Priori SG Napolitano C Memmi M Colombi B Drago F Gasparini M. Clinical and molecular characterization of patients with catecholaminergic polymorphic ventricular tachycardia. Circulation. (2002) 106:69–74. 10.1161/01.CIR.0000020013.73106.D8 12093772

[116] Blancard M Touat-Hamici Z Aguilar-Sanchez Y Yin L Vaksmann G Roux-Buisson N. A type 2 ryanodine receptor variant in the helical domain 2 associated with an impairment of the adrenergic response. J Pers Med. (2021) 11:579. 10.3390/jpm11060579 34202968 PMC8235491

[117] Hirose S Murayama T Tetsuo N Hoshiai M Kise H Yoshinaga M. Loss-of-function mutations in cardiac ryanodine receptor channel cause various types of arrhythmias including long QT syndrome. Europace. (2021) 24:497–510. 10.1093/europace/euab250 34661651

[118] di Barletta MR Viatchenko-Karpinski S Nori A Memmi M Terentyev D Turcato F. Clinical phenotype and functional characterization of CASQ2 mutations associated with catecholaminergic polymorphic ventricular tachycardia. Circulation. (2006) 114:1012–9. 10.1161/CIRCULATIONAHA.106.623793 16908766

[119] Faggioni M Kryshtal DO Knollmann BC. Calsequestrin mutations and catecholaminergic polymorphic ventricular tachycardia. Pediatr Cardiol. (2012) 33:959–67. 10.1007/s00246-012-0256-1 22421959 PMC3393815

[120] Zhang L Kelley J Schmeisser G Kobayashi YM Jones LR. Complex formation between junctin, triadin, calsequestrin, and the ryanodine receptor. Proteins of the cardiac junctional sarcoplasmic reticulum membrane. J Biol Chem. (1997) 272:23389–97. 10.1074/jbc.272.37.23389 9287354

[121] Chopra N Yang T Asghari P Moore ED Huke S Akin B. Ablation of triadin causes loss of cardiac Ca2+ release units, impaired excitation-contraction coupling, and cardiac arrhythmias. Proc Natl Acad Sci USA. (2009) 106:7636–41. 10.1073/pnas.0902919106 19383796 PMC2678594

[122] Rabbani B Khorgami M Dalili M Zamani N Mahdieh N Gollob MH. Novel cases of pediatric sudden cardiac death secondary to TRDN mutations presenting as long QT syndrome at rest and catecholaminergic polymorphic ventricular tachycardia during exercise: the TRDN arrhythmia syndrome. Am J Med Genet a. (2021) 185:3433–45. 10.1002/ajmg.a.62464 34415104

[123] Clemens DJ Tester DJ Marty I Ackerman MJ. Phenotype-guided whole genome analysis in a patient with genetically elusive long QT syndrome yields a novel TRDN-encoded triadin pathogenetic substrate for triadin knockout syndrome and reveals a novel primate-specific cardiac TRDN transcript. Heart Rhythm. (2020) 17:1017–24. 10.1016/j.hrthm.2020.01.012 32402482

[124] Ben-Johny M Yue DT. Calmodulin regulation (calmodulation) of voltage-gated calcium channels. J Gen Physiol. (2014) 143:679–92. 10.1085/jgp.201311153 24863929 PMC4035741

[125] Brohus M Arsov T Wallace DA Jensen HH Nyegaard M Crotti L. Infanticide vs. inherited cardiac arrhythmias. Europace. (2021) 23:441–50. 10.1093/europace/euaa272 33200177 PMC7947592

[126] Choi JI Wang C Thomas MJ Pitt GS. Alpha1-Syntrophin variant identified in drug-induced long QT syndrome increases late sodium current. PLoS ONE. (2016) 11:e152355. 10.1371/journal.pone.0152355 27028743 PMC4814026

[127] Campuzano O Fernandez-Falgueras A Iglesias A Brugada R. Brugada syndrome and PKP2: evidences and uncertainties. Int J Cardiol. (2016) 214:403–5. 10.1016/j.ijcard.2016.03.194 27085656

[128] Li J Chen K Zhu R Zhang M. Structural basis underlying strong interactions between ankyrins and spectrins. J Mol Biol. (2020) 432:3838–50. 10.1016/j.jmb.2020.04.023 32353364

[129] Steinfurt J Bezzina CR Biermann J Staudacher D Marschall C Trolese L. Two siblings with early repolarization syndrome: clinical and genetic characterization by whole-exome sequencing. Europace. (2021) 23:775–80. 10.1093/europace/euaa357 33324992 PMC8139820

[130] Prasad V Bodi I Meyer JW Wang Y Ashraf M Engle SJ. Impaired cardiac contractility in mice lacking both the AE3 Cl-/HCO3- exchanger and the NKCC1 Na+-K+-2Cl- cotransporter: effects on Ca2+ handling and protein phosphatases. J Biol Chem. (2008) 283:31303–14. 10.1074/jbc.M803706200 18779325 PMC2581574

[131] Visser M van der Heijden JF Doevendans PA Loh P Wilde AA Hassink RJ. Idiopathic ventricular fibrillation: the struggle for definition, diagnosis, and follow-up. Circ Arrhythm Electrophysiol. (2016) 9:e003817. 10.1161/CIRCEP.115.003817 27103090

[132] Marsman RF Barc J Beekman L Alders M Dooijes D van den Wijngaard A. A mutation in CALM1 encoding calmodulin in familial idiopathic ventricular fibrillation in childhood and adolescence. J Am Coll Cardiol. (2014) 63:259–66. 10.1016/j.jacc.2013.07.091 24076290

[133] Alders M Koopmann TT Christiaans I Postema PG Beekman L Tanck MW. Haplotype-sharing analysis implicates chromosome 7q36 harboring DPP6 in familial idiopathic ventricular fibrillation. Am J Hum Genet. (2009) 84:468–76. 10.1016/j.ajhg.2009.02.009 19285295 PMC2667995

[134] Roston TM Sanatani S Chen SR. Suppression-of-function mutations in the cardiac ryanodine receptor: emerging evidence for a novel arrhythmia syndrome? Heart Rhythm. (2017) 14:108–9. 10.1016/j.hrthm.2016.11.004 27818320

[135] Walsh MA Stuart AG Schlecht HB James AF Hancox JC Newbury-Ecob RA. Compound heterozygous triadin mutation causing cardiac arrest in two siblings. Pacing Clin Electrophysiol. (2016) 39:497–501. 10.1111/pace.12813 26768964

[136] Blancard M Debbiche A Kato K Cardin C Sabrina G Gandjbakhch E. An African loss-of-function CACNA1C variant p.T1787M associated with a risk of ventricular fibrillation. Sci Rep. (2018) 8:14619. 10.1038/s41598-018-32867-4 30279520 PMC6168548

[137] Akai J Makita N Sakurada H Shirai N Ueda K Kitabatake A. A novel SCN5A mutation associated with idiopathic ventricular fibrillation without typical ECG findings of Brugada syndrome. Febs Lett. (2000) 479:29–34. 10.1016/S0014-5793(00)01875-5 10940383

[138] Priori SG Blomstrom-Lundqvist C Mazzanti A Blom N Borggrefe M Camm J. 2015 ESC guidelines for the management of patients with ventricular arrhythmias and the prevention of sudden cardiac death: the task force for the management of patients with ventricular arrhythmias and the prevention of sudden cardiac death of the European society of cardiology (ESC) endorsed by: association for European paediatric and congenital cardiology (AEPC). Europace. (2015) 17:1601–87. 10.1093/eurheartj/ehv316 26318695

[139] Jerng HH Qian Y Pfaffinger PJ. Modulation of Kv4.2 channel expression and gating by dipeptidyl peptidase 10 (DPP10). Biophys J. (2004) 87:2380–96. 10.1529/biophysj.104.042358 15454437 PMC1304660

[140] Seikel E Trimmer JS. Convergent modulation of Kv4.2 channel alpha subunits by structurally distinct DPPX and KChIP auxiliary subunits. Biochemistry. (2009) 48:5721–30. 10.1021/bi802316m 19441798 PMC2733230

[141] Soh H Goldstein SA. I SA channel complexes include four subunits each of DPP6 and Kv4.2. J Biol Chem. (2008) 283:15072–7. 10.1074/jbc.M706964200 18364354 PMC2397469

[142] Ten SJ Postema PG Boekholdt SM Tan HL van der Heijden JF de Groot NM. Detailed characterization of familial idiopathic ventricular fibrillation linked to the DPP6 locus. Heart Rhythm. (2016) 13:905–12. 10.1016/j.hrthm.2015.12.006 26681609

[143] Conte G Giudicessi JR Ackerman MJ. Idiopathic ventricular fibrillation: the ongoing quest for diagnostic refinement. Europace. (2021) 23:4–10. 10.1093/europace/euaa211 33038214

[144] Bezzina C Veldkamp MW van Den Berg MP Postma AV Rook MB Viersma JW. A single Na(+) channel mutation causing both long-QT and Brugada syndromes. Circ Res. (1999) 85:1206–13. 10.1161/01.RES.85.12.1206 10590249

[145] Risgaard B Jabbari R Refsgaard L Holst AG Haunso S Sadjadieh A. High prevalence of genetic variants previously associated with Brugada syndrome in new exome data. Clin Genet. (2013) 84:489–95. 10.1111/cge.12126 23414114

[146] Priori SG Napolitano C Schwartz PJ. Low penetrance in the long-QT syndrome: clinical impact. Circulation. (1999) 99:529–33. 10.1161/01.CIR.99.4.529 9927399

[147] Clatot J Neyroud N Cox R Souil C Huang J Guicheney P. Inter-regulation of kv4.3 and voltage-gated sodium channels underlies predisposition to cardiac and neuronal channelopathies. Int J Mol Sci. (2020) 21:5057. 10.3390/ijms21145057 32709127 PMC7404392

[148] Chen L Kass RS. A-kinase anchoring protein 9 and IKs channel regulation. J Cardiovasc Pharmacol. (2011) 58:413–59. 10.1097/FJC.0b013e318232c80c 21885989 PMC3226347

[149] Zhang R Chen F Yu H Gao L Yin X Dong Y. The genetic variation rs12143842 in NOS1AP increases idiopathic ventricular tachycardia risk in Chinese Han populations. Sci Rep. (2017) 7:8356. 10.1038/s41598-017-08548-z 28827735 PMC5567283

[150] Adler A Topaz G Heller K Zeltser D Ohayon T Rozovski U. Fever-induced Brugada pattern: How common is it and what does it mean? Heart Rhythm. (2013) 10:1375–82. 10.1016/j.hrthm.2013.07.030 23872691 PMC3832740

[151] Mascia G Arbelo E Hernandez-Ojeda J Solimene F Brugada R Brugada J. Brugada syndrome and exercise practice: current knowledge, shortcomings and open questions. Int J Sports Med. (2017) 38:573–81. 10.1055/s-0043-107240 28625016

[152] Iglesias DG Rubin J Perez D Moris C Calvo D. Insights for stratification of risk in Brugada syndrome. Eur Cardiol. (2019) 14:45–9. 10.15420/ecr.2018.31.2 31131036 PMC6523056

[153] Cavalli G Heard E. Advances in epigenetics link genetics to the environment and disease. Nature. (2019) 571:489–99. 10.1038/s41586-019-1411-0 31341302

[154] Sommariva E D'Alessandra Y Farina FM Casella M Cattaneo F Catto V. MiR-320a as a potential novel circulating biomarker of arrhythmogenic CardioMyopathy. Sci Rep. (2017) 7:4802. 10.1038/s41598-017-05001-z 28684747 PMC5500514

[155] Kiehne N Kauferstein S. Mutations in the SCN5A gene: evidence for a link between long QT syndrome and sudden death? Forensic Sci Int Genet. (2007) 1:170–4. 10.1016/j.fsigen.2007.01.009 19083750

[156] Ricci MT Menegon S Vatrano S Mandrile G Cerrato N Carvalho P. SCN1B gene variants in Brugada syndrome: a study of 145 SCN5A-negative patients. Sci Rep. (2014) 4:6470. 10.1038/srep06470 25253298 PMC5377327

[157] Hedley PL Carlsen AL Christiansen KM Kanters JK Behr ER Corfield VA. MicroRNAs in cardiac arrhythmia: DNA sequence variation of MiR-1 and MiR-133A in long QT syndrome. Scand J Clin Lab Invest. (2014) 74:485–91. 10.3109/00365513.2014.905696 24809446 PMC4196592

[158] Gong Q Stump MR Zhou Z. Upregulation of functional Kv11.1a isoform expression by modified U1 small nuclear RNA. Gene. (2018) 641:220–5. 10.1016/j.gene.2017.10.063 29066300 PMC5755592

[159] Gong Q Stump MR Deng V Zhang L Zhou Z. Identification of Kv11.1 isoform switch as a novel pathogenic mechanism of long-QT syndrome. Circ Cardiovasc Genet. (2014) 7:482–90. 10.1161/CIRCGENETICS.114.000586 25028483 PMC4180443

[160] Lizotte E Junttila MJ Dube MP Hong K Benito B DE Zutter M. Genetic modulation of brugada syndrome by a common polymorphism. J Cardiovasc Electrophysiol. (2009) 20:1137–41. 10.1111/j.1540-8167.2009.01508.x 19549036

[161] Viswanathan PC Benson DW Balser JR. A common SCN5A polymorphism modulates the biophysical effects of an SCN5A mutation. J Clin Invest. (2003) 111:341–6. 10.1172/JCI200316879 12569159 PMC151865

[162] Shinlapawittayatorn K Du XX Liu H Ficker E Kaufman ES Deschenes I. A common SCN5A polymorphism modulates the biophysical defects of SCN5A mutations. Heart Rhythm. (2011) 8:455–62. 10.1016/j.hrthm.2010.11.034 21109022 PMC3050092

[163] Ye B Valdivia CR Ackerman MJ Makielski JC. A common human SCN5A polymorphism modifies expression of an arrhythmia causing mutation. Physiol Genomics. (2003) 12:187–93. 10.1152/physiolgenomics.00117.2002 12454206

[164] Shinlapawittayatorn K Dudash LA Du XX Heller L Poelzing S Ficker E. A novel strategy using cardiac sodium channel polymorphic fragments to rescue trafficking-deficient SCN5A mutations. Circ Cardiovasc Genet. (2011) 4:500–9. 10.1161/CIRCGENETICS.111.960633 21840964 PMC3197758

[165] Gouas L Nicaud V Berthet M Forhan A Tiret L Balkau B. Association of KCNQ1, KCNE1, KCNH2 and SCN5A polymorphisms with QTc interval length in a healthy population. Eur J Hum Genet. (2005) 13:1213–22. 10.1038/sj.ejhg.5201489 16132053

[166] Tsukakoshi T Lin L Murakami T Shiono J Izumi I Horigome H. Persistent QT prolongation in a child with gitelman syndrome and SCN5A H558R polymorphism. Int Heart J. (2018) 59:1466–8. 10.1536/ihj.17-686 30305584

[167] Li X. Genomic imprinting is a parental effect established in mammalian germ cells. Curr Top Dev Biol. (2013) 102:35–59. 10.1016/B978-0-12-416024-8.00002-7 23287029

[168] Jimenez J Rentschler SL. Transcriptional and epigenetic regulation of cardiac electrophysiology. Pediatr Cardiol. (2019) 40:1325–30. 10.1007/s00246-019-02160-w 31346662 PMC6785575

[169] Rao SS Huntley MH Durand NC Stamenova EK Bochkov ID Robinson JT. A 3D map of the human genome at kilobase resolution reveals principles of chromatin looping. Cell. (2014) 159:1665–80. 10.1016/j.cell.2014.11.021 25497547 PMC5635824

[170] Nothjunge S Nuhrenberg TG Gruning BA Doppler SA Preissl S Schwaderer M. DNA methylation signatures follow preformed chromatin compartments in cardiac myocytes. Nat Commun. (2017) 8:1667. 10.1038/s41467-017-01724-9 29162810 PMC5698409

[171] Monk D Mackay D Eggermann T Maher ER Riccio A. Genomic imprinting disorders: lessons on how genome, epigenome and environment interact. Nat Rev Genet. (2019) 20:235–48. 10.1038/s41576-018-0092-0 30647469

[172] Smilinich NJ Day CD Fitzpatrick GV Caldwell GM Lossie AC Cooper PR. A maternally methylated CpG island in KvLQT1 is associated with an antisense paternal transcript and loss of imprinting in Beckwith-Wiedemann syndrome. Proc Natl Acad Sci USA. (1999) 96:8064–9. 10.1073/pnas.96.14.8064 10393948 PMC22188

[173] Korostowski L Raval A Breuer G Engel N. Enhancer-driven chromatin interactions during development promote escape from silencing by a long non-coding RNA. Epigenetics Chromatin. (2011) 4:21. 10.1186/1756-8935-4-21 22085535 PMC3228667

[174] Singh P Wu X Lee DH Li AX Rauch TA Pfeifer GP. Chromosome-wide analysis of parental allele-specific chromatin and DNA methylation. Mol Cell Biol. (2011) 31:1757–70. 10.1128/MCB.00961-10 21321082 PMC3126330

[175] Itoh H Berthet M Fressart V Denjoy I Maugenre S Klug D. Asymmetry of parental origin in long QT syndrome: Preferential maternal transmission of KCNQ1 variants linked to channel dysfunction. Eur J Hum Genet. (2016) 24:1160–6. 10.1038/ejhg.2015.257 26669661 PMC4970673

[176] Jiang Y Du W Chu Q Qin Y Tuguzbaeva G Wang H. Downregulation of long non-coding RNA kcnq1ot1: an important mechanism of arsenic trioxide-induced long QT syndrome. Cell Physiol Biochem. (2018) 45:192–202. 10.1159/000486357 29339628

[177] Shan H Zhang Y Cai B Chen X Fan Y Yang L. Upregulation of microRNA-1 and microRNA-133 contributes to arsenic-induced cardiac electrical remodeling. Int J Cardiol. (2013) 167:2798–805. 10.1016/j.ijcard.2012.07.009 22889704

[178] Zhang Y Li G. Advances in technologies for 3D genomics research. Sci China Life Sci. (2020) 63:811–24. 10.1007/s11427-019-1704-2 32394244

[179] Schwarzer W Abdennur N Goloborodko A Pekowska A Fudenberg G Loe-Mie Y. Two independent modes of chromatin organization revealed by cohesin removal. Nature. (2017) 551:51–6. 10.1038/nature24281 29094699 PMC5687303

[180] Woodcock CL Ghosh RP. Chromatin higher-order structure and dynamics. Cold Spring Harb Perspect Biol. (2010) 2:a596. 10.1101/cshperspect.a000596 20452954 PMC2857170

[181] Stadhouders R Filion GJ Graf T. Transcription factors and 3D genome conformation in cell-fate decisions. Nature. (2019) 569:345–54. 10.1038/s41586-019-1182-7 31092938

[182] Seitan VC Faure AJ Zhan Y McCord RP Lajoie BR Ing-Simmons E. Cohesin-based chromatin interactions enable regulated gene expression within preexisting architectural compartments. Genome Res. (2013) 23:2066–77. 10.1101/gr.161620.113 24002784 PMC3847776

[183] Sofueva S Yaffe E Chan WC Georgopoulou D Vietri RM Mira-Bontenbal H. Cohesin-mediated interactions organize chromosomal domain architecture. Embo J. (2013) 32:3119–29. 10.1038/emboj.2013.237 24185899 PMC4489921

[184] Ouimette JF Rougeulle C Veitia RA. Three-dimensional genome architecture in health and disease. Clin Genet. (2019) 95:189–98. 10.1111/cge.13219 29377081

[185] van den Boogaard M Smemo S Burnicka-Turek O Arnolds DE van de Werken HJ Klous P. A common genetic variant within SCN10A modulates cardiac SCN5A expression. J Clin Invest. (2014) 124:1844–52. 10.1172/JCI73140 24642470 PMC3973109

[186] van den Boogaard M Wong LY Tessadori F Bakker ML Dreizehnter LK Wakker V. Genetic variation in T-box binding element functionally affects SCN5A/SCN10A enhancer. J Clin Invest. (2012) 122:2519–30. 10.1172/JCI62613 22706305 PMC3386824

[187] Koopmann TT Adriaens ME Moerland PD Marsman RF Westerveld ML Lal S. Genome-wide identification of expression quantitative trait loci (eQTLs) in human heart. PLoS ONE. (2014) 9:e97380. 10.1371/journal.pone.0097380 24846176 PMC4028258

[188] van Ouwerkerk AF Bosada FM van Duijvenboden K Hill MC Montefiori LE Scholman KT. Identification of atrial fibrillation associated genes and functional non-coding variants. Nat Commun. (2019) 10:4755. 10.1038/s41467-019-12721-5 31628324 PMC6802215

[189] Montefiori LE Sobreira DR Sakabe NJ Aneas I Joslin AC Hansen GT. A promoter interaction map for cardiovascular disease genetics. Elife. (2018) 7:e35788. 10.7554/eLife.35788.103 29988018 PMC6053306

[190] Lahrouchi N Tadros R Crotti L Mizusawa Y Postema PG Beekman L. Transethnic genome-wide association study provides insights in the genetic architecture and heritability of long QT syndrome. Circulation. (2020) 142:324–38. 10.1161/CIRCULATIONAHA.120.045956 32429735 PMC7382531

[191] Gacita AM Fullenkamp DE Ohiri J Pottinger T Puckelwartz MJ Nobrega MA. Genetic variation in enhancers modifies cardiomyopathy gene expression and progression. Circulation. (2021) 143:1302–16. 10.1161/CIRCULATIONAHA.120.050432 33478249 PMC8009836

